# Research Progress on the Construction of Pickering Emulsion and Gel Systems and Their Applications in the Food Industry

**DOI:** 10.3390/foods15203634

**Published:** 2026-10-13

**Authors:** Huiqiao Zhou, Xing Zhang, Xin Li

**Affiliations:** 1 State Key Laboratory of Food Science and Resources, Nanchang University, Nanchang 330047, China; 18720936727@163.com; 2 School of Food Science and Technology, Nanchang University, Nanchang 330047, China; 3 Faculty of Medicine, Macau University of Science and Technology, Macau 999078, China; 4 Jiangxi Medicine Academy of Nutrition and Health Management, The First Affiliated Hospital, Jiangxi Medical College, Nanchang University, Nanchang 330006, China

**Keywords:** Pickering emulsion gels, stabilization mechanisms, food-grade particles, food applications

## Abstract

Traditional synthetic emulsifiers have raised increasing concerns due to potential health risks and environmental issues. Pickering emulsions stabilized by natural solid particles have emerged as an important alternative. Such emulsions exploit the irreversible adsorption of particles at oil–water interfaces to form a stable barrier, combining stability with biocompatibility, making them suitable for constructing food-grade soft solid materials. This review summarizes the stabilization mechanisms, construction strategies, and application progress of Pickering emulsion gels in food. First, the irreversible adsorption of solid particles at oil–water interfaces and the formation mechanisms of gel networks are described, with an analysis of the effects of particle properties and environmental conditions on system stability. Subsequently, the types of food-grade stabilizing particles are introduced, including protein-based, polysaccharide-based, and composite particles. Then, the formation and stabilization mechanisms of Pickering emulsion gel systems, as well as their applications in food domains such as fat replacers, bioactive substance delivery, 3D-printed foods, and active packaging, are discussed. In addition, key challenges in the industrial translation of Pickering emulsion gels are discussed from multiple perspectives. Future research directions include the development of green, intelligent, and multifunctional Pickering emulsion gels, achieving precision nutrition delivery through programmed sequential release, and overcoming freeze–thaw stability bottlenecks to advance industrialized applications.

## 1. Introduction

The modern food industry relies heavily on emulsifiers to maintain the texture and appearance of processed foods [1,2,3]. Although synthetic emulsifiers are generally recognized as safe when used within regulatory limits, accumulating toxicological evidence in recent years has raised new concerns regarding their long-term health effects, particularly with respect to gut microbiota homeostasis and low-grade inflammation [4,5]. Therefore, replacing synthetic emulsifiers with natural solid particles has become an important research direction [4]. On the other hand, most studies remain confined to animal models or high-dose exposure conditions, and the relevance of these findings to human health has yet to reach a consensus. This ongoing controversy provides a timely opportunity for the development of Pickering emulsion systems stabilized by natural particles—systems that may not only circumvent the aforementioned potential risks but also offer the benefits of environmental friendliness and multifunctional integration.

The concept of particle-stabilized interfaces was first reported by Ramsden [6] and later systematically characterized by Pickering [7], who identified irreversible particle adsorption as the key to exceptional emulsion stability. These solid-stabilized systems, subsequently termed Pickering emulsions, offer distinct advantages over traditional emulsions, including lower toxicity, reduced emulsifier usage, and environmental friendliness [6,7,8]. More recently, the gelation of Pickering emulsions has gained considerable attention. Pickering emulsion gels are viscoelastic colloidal materials formed by incorporating gelling agents, creating three-dimensional networks that enhance both stability and functionality. Depending on the primary gelling agent, they can be classified as protein-, polysaccharide-, or composite-based emulsion gels [9]. These systems synergistically combine the benefits of emulsions and gels, markedly improving storage stability and the controlled release of lipophilic components, while effectively simulating the texture and sensory attributes of full-fat foods in low-fat products [10,11,12].

Recent reviews on food-grade Pickering emulsions have mainly focused on particle design, interfacial behavior, stabilization mechanisms, and their applications in different food systems, whereas reviews on Pickering emulsion gels have increasingly emphasized gel-forming materials, structural and rheological properties, stability, and emerging food applications. In contrast, this review links Pickering emulsion stabilization with subsequent gel formation by connecting particle characteristics and interfacial stabilization with gel network formation and rheological behavior, while further distinguishing emulsion stability from emulsion gel stability. The discussion is further extended to the relationships between the structural, interfacial, and rheological characteristics of Pickering emulsion gels and their functional performance in fat replacement, bioactive delivery, 3D printing, and food preservation. The practical translation of these applications and the associated industrial challenges are also discussed.

## 2. Stabilization Mechanisms and Influencing Factors of Pickering Emulsions

### 2.1. Stabilization Mechanisms

Pickering emulsions are stabilized by solid particles strongly anchored at the oil–water interface. Unlike surfactants, which can rapidly adsorb and desorb, interfacial particles generally require substantially more energy for detachment. For a spherical particle, the detachment energy is

Δ*E* = *πr*^2^*γ*(1 ± |cos *θ*|)^2^ (1)

where *r* is the particle radius, *γ* is the oil–water interfacial tension, and *θ* is the three-phase contact angle. The sign depends on the phase from which the particle is detached. The equation shows that the detachment energy increases with particle size and oil–water interfacial tension and reaches its maximum when the contact angle approaches 90°. Kumar et al. calculated that for silica nanoparticles with a diameter of approximately 15 nm at a contact angle close to 90°, the detachment energy can reach approximately 8943 *k*_B_*T*, far exceeding the characteristic thermal energy *k*_B_*T* and the typical adsorption energy of surfactant molecules (approximately 10 *k*_B_*T*). This means that the energy barrier to particle detachment is nearly four orders of magnitude greater than thermal fluctuations, leading to near-irreversible adsorption behavior. Upon stable adsorption at the oil–water interface, the particles reduce the interfacial tension by lowering the interfacial free energy, thereby decreasing the thermodynamic driving force for droplet coalescence. Simultaneously, the interfacial particle layer forms a rigid mechanical barrier that prevents direct droplet contact and coalescence, thus delaying droplet aggregation and macroscopic phase separation of the oil and water phases [13].

Furthermore, excess particles may form three-dimensional network structures in the continuous phase, enhancing kinetic stability by increasing system viscosity and restricting droplet mobility [14,15]. Finally, electrostatic repulsion between particles or droplets also plays an important auxiliary stabilizing role. By adjusting the pH or ionic strength of the system, the surface charge density of particles can be modified, enhancing electrostatic repulsion between particles, preventing droplet aggregation, and further improving the physical stability of the emulsion [16].

### 2.2. Key Factors Affecting Stability

Factors affecting the stability of Pickering emulsions include particle properties (particle wettability, size, shape, surface charge, surface roughness, etc.), system composition factors (particle concentration, oil phase properties, oil–water phase ratio), and environmental conditions (pH, ionic strength, temperature).

#### 2.2.1. Particle Properties

##### Particle Wettability

**Figure 1:**
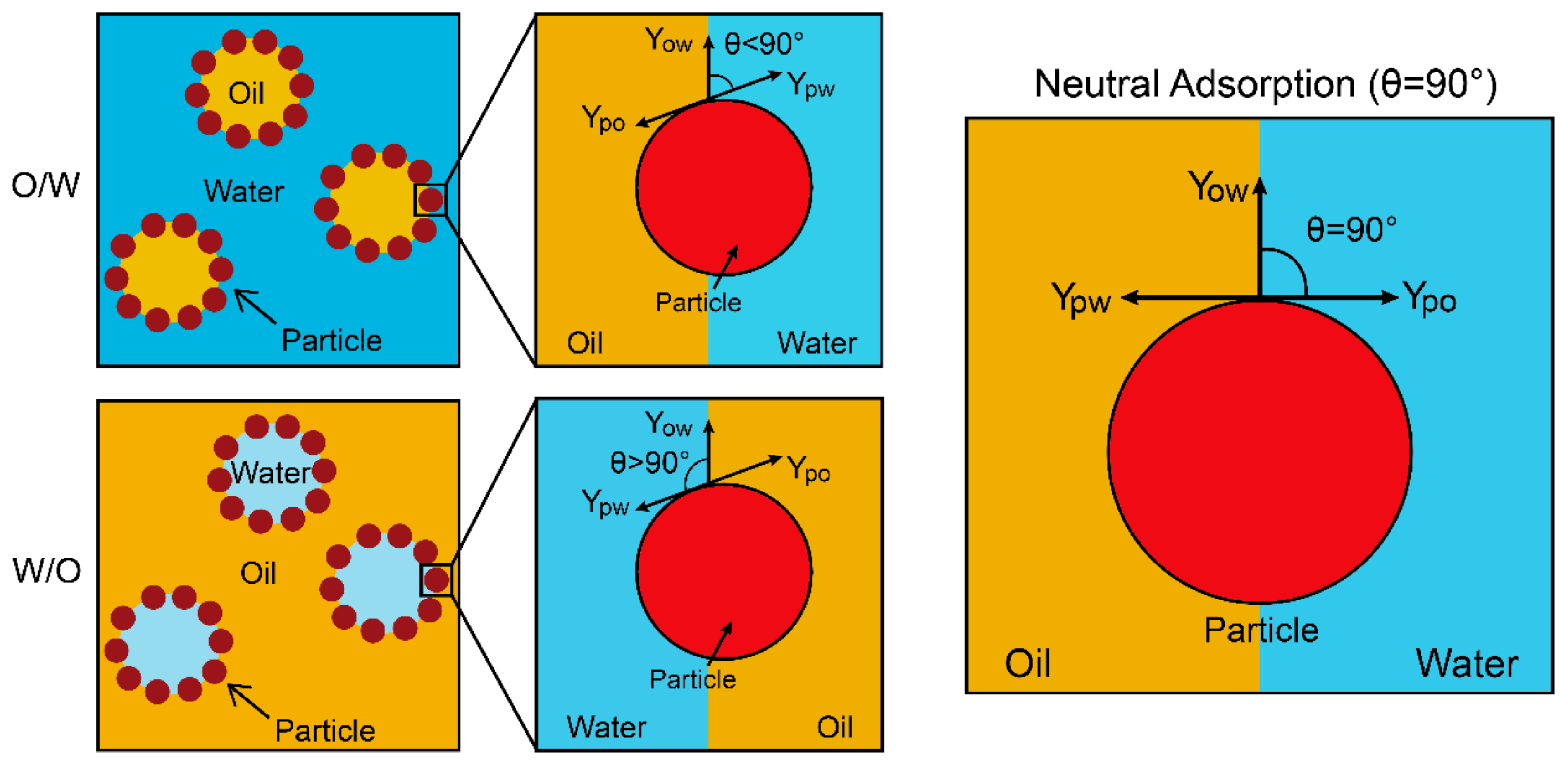
Schematic diagram of O/W and W/O Pickering emulsion microstructures.

Particles must be partially wetted by both liquid phases to stabilize a Pickering emulsion. Wettability is characterized by the three-phase contact angle, θ, measured through the aqueous phase. Hydrophilic particles with θ < 90° generally favor O/W emulsions, whereas hydrophobic particles with θ > 90° favor W/O emulsions (Figure 1) [17]. As discussed in Section 2.1, attachment is strongest near θ = 90°. Deviation lowers the smaller detachment barrier, facilitating desorption and potentially weakening stability.

Contact-angle measurements are less straightforward for food-grade colloidal particles than for ideal rigid particles. Tablet compression yields apparent values that can be affected by particle size, surface heterogeneity, roughness and porosity [18,19]. In recent years, improved methods that more closely approach the real interfacial configuration have emerged. Xu et al. fixed interfacial particles by UV polymerization and used scanning electron microscopy to determine their position and contact angle [20]. Zhou et al. used confocal microscopy to directly measure particle contact angles and interfacial coverage in situ [21]. Nevertheless, soft particles may deform and extend molecular chains into both phases, making a single static contact angle insufficient to describe their interfacial behavior [19]. Further in situ characterization under realistic food conditions is therefore needed. Combining microstructural observations, adsorption measurements and interfacial rheology would provide a more complete assessment of particle stabilization than contact angle alone.

##### Particle Shape and Size

Particle shape is also a key factor affecting the stability of Pickering emulsions. The detachment energies of particles with different shapes at the oil–water interfaces differ significantly: under the same surface properties, the order of detachment energy magnitude is spherical particles < rod-shaped particles < disk-shaped particles. This is because anisotropic particles typically have larger aspect ratios, enabling them to cover a larger interfacial area and form high-strength crosslinked network structures, thereby significantly enhancing emulsion stability [22]. Meanwhile, the specific surface area of non-spherical particles is generally larger than that of spherical particles, giving them stronger interfacial adsorption capacity and further facilitating emulsion stabilization [23]. Madivala et al. further demonstrated that the higher the particle aspect ratio, the greater the interfacial packing density and viscoelastic modulus, the stronger the emulsion stability, and the more oil phase that can be emulsified [24]. Particle size can also influence interfacial coverage and emulsion stability, but its effect is system-dependent. In some systems, smaller particles may provide denser interfacial coverage and enhance stability, whereas particle adsorption kinetics, surface chemistry, and interparticle interactions can modify this relationship.

##### Particle Surface Charge

Particle charge must balance dispersion stability against interfacial assembly. When the adsorption ability of particles at the interface is weak, for instance due to poor wetting properties, the repulsive force between particles of the same charge may still restrict the aggregation of particles and droplets [17]. However, an excessively high particle charge may impede rapid adsorption at the interface due to strong electrostatic repulsion; the extent of this effect depends on particle wettability, surface chemistry, and the balance between electrostatic repulsion and interfacial adsorption [25]. Therefore, an appropriate surface charge is needed to ensure stable dispersion of particles in solution while facilitating rapid adsorption and dense packing at the interface; however, the optimal charge density is system-dependent and should be determined based on the particle material, solution conditions, and desired interfacial properties.

##### Particle Roughness

The effect of particle surface roughness on the stability of Pickering emulsions is not simply a matter of promotion or inhibition, but rather depends on multiple factors, including the wetting regime and the scale of roughness. Early work by Vignati et al. found that rough surfaces weakened the emulsifying ability of particles [26]. However, San-Miguel and Behrens, by precisely tuning roughness without altering surface chemistry, revealed a nonmonotonic effect: in the Wenzel wetting regime (where the liquid fully penetrates the rough structures), roughness enhanced emulsion stability by increasing contact angle hysteresis and strengthening the interfacial pinning of particles; in contrast, when the wetting regime transitioned to the Cassie–Baxter state (where fluid is trapped between the liquid and the rough surface), stability was reduced [27].

These contrasting observations indicate that the influence of roughness is governed by the actual wetting state. The particle material determines its intrinsic wettability, while roughness (both amplitude and topography) further modulates the wetting behavior. When rough structures effectively pin the three-phase contact line, contact angle hysteresis increases, particles are firmly anchored at the interface, and emulsion stability is enhanced. Conversely, if the rough structures do not provide sufficient pinning points, the three-phase contact line tends to move more readily, resulting in smaller contact angle hysteresis, easier particle desorption, and decreased stability. Therefore, when utilizing roughness to modulate emulsion stability, it is essential to consider the particle material, the roughness scale, and the target wetting regime in combination to achieve optimal stabilization.

#### 2.2.2. System Composition Factors

##### Particle Concentration

**Figure 2:**
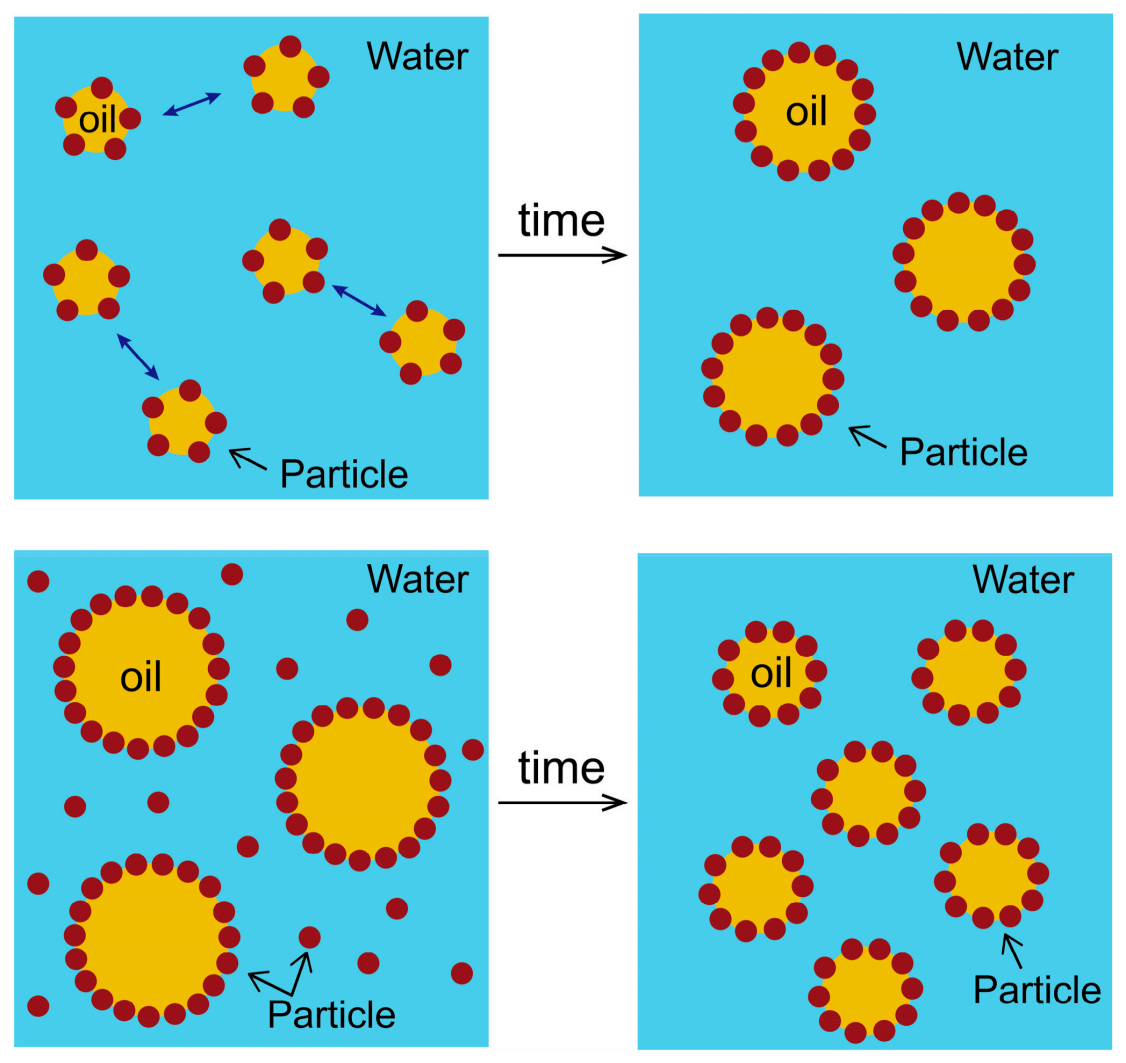
Effect of particle concentration on droplet size in Pickering emulsions.

Within a certain range of particle concentration, increasing the concentration of solid particles will reduce the droplet size of the emulsion, thereby improving emulsion stability. The reason is as follows: when particle concentration is insufficient, the interfacial area newly generated during emulsification exceeds the area that particles can stably cover, causing droplets to merge until the interfacial area reduces to the extent that particles can just cover, often resulting in instability; conversely, when particle concentration increases and can stabilize a larger interfacial area, droplet size decreases (Figure 2) [28]. At high particle concentrations, compared with the oil/water interfacial area generated during emulsification, an excess of particles leads to two situations: 1) droplets of any size formed during emulsification are immediately stabilized, resulting in non-uniform sizes; 2) droplet size remains constant but excess particles in the continuous phase form a network in the continuous phase, improving emulsion stability [29].

##### Oil Phase Properties

Oil phase properties have an important influence on the preparation of Pickering emulsions; the main affecting parameters are oil phase viscosity and polarity. Oil polarity can alter particle wettability and thus determine the emulsion type. For example, non-polar oils (such as hydrocarbons) can make silica particles with moderate wettability appear more hydrophilic, tending to stabilize O/W emulsions, while polar oils (such as esters and alcohols) make the same particles appear more hydrophobic, tending to stabilize W/O emulsions. Oil polarity also affects the adsorption and stabilization energy of different particle types. Thickett et al. demonstrated that, compared to polar oils, the stabilization energy generated by graphite oxide particle adsorption is higher in non-polar oils, resulting in more stable emulsions formed in non-polar oils [30]. Oil phase viscosity also affects droplet size and emulsion stability. Fournier et al. noted that the oil type has a significant influence on the stability of emulsions stabilized by cellulose nanocrystals. Under the same emulsification conditions, low-viscosity decane more easily forms stable Pickering emulsions than high-viscosity corn oil. Oil viscosity is a limiting factor for particle adsorption at the oil/water interface, as it slows down particle diffusion and adsorption rates [31].

##### Oil–Water Ratio

The oil–water ratio also affects the stability of Pickering emulsions. For O/W-type Pickering emulsions, increasing the oil–water ratio within a certain range causes the emulsion particle size to increase as emulsion stability deteriorates. This is because for O/W emulsions, when the solid particle amount is fixed and the oil phase proportion increases, the amount of solid particles adsorbed per unit area decreases. If the oil droplet surface is not completely covered by solid particles to form a stable solid interfacial film, droplets will attract each other due to van der Waals forces and undergo a degree of flocculation or aggregation, deteriorating emulsion stability [32]. Whether for O/W- or W/O-type Pickering emulsions, neither the smallest nor the largest oil–water ratio leads to the best stability; both types of emulsions have an optimal oil–water ratio at which stability is best [33].

#### 2.2.3. Environmental Conditions

##### pH

**Figure 3:**
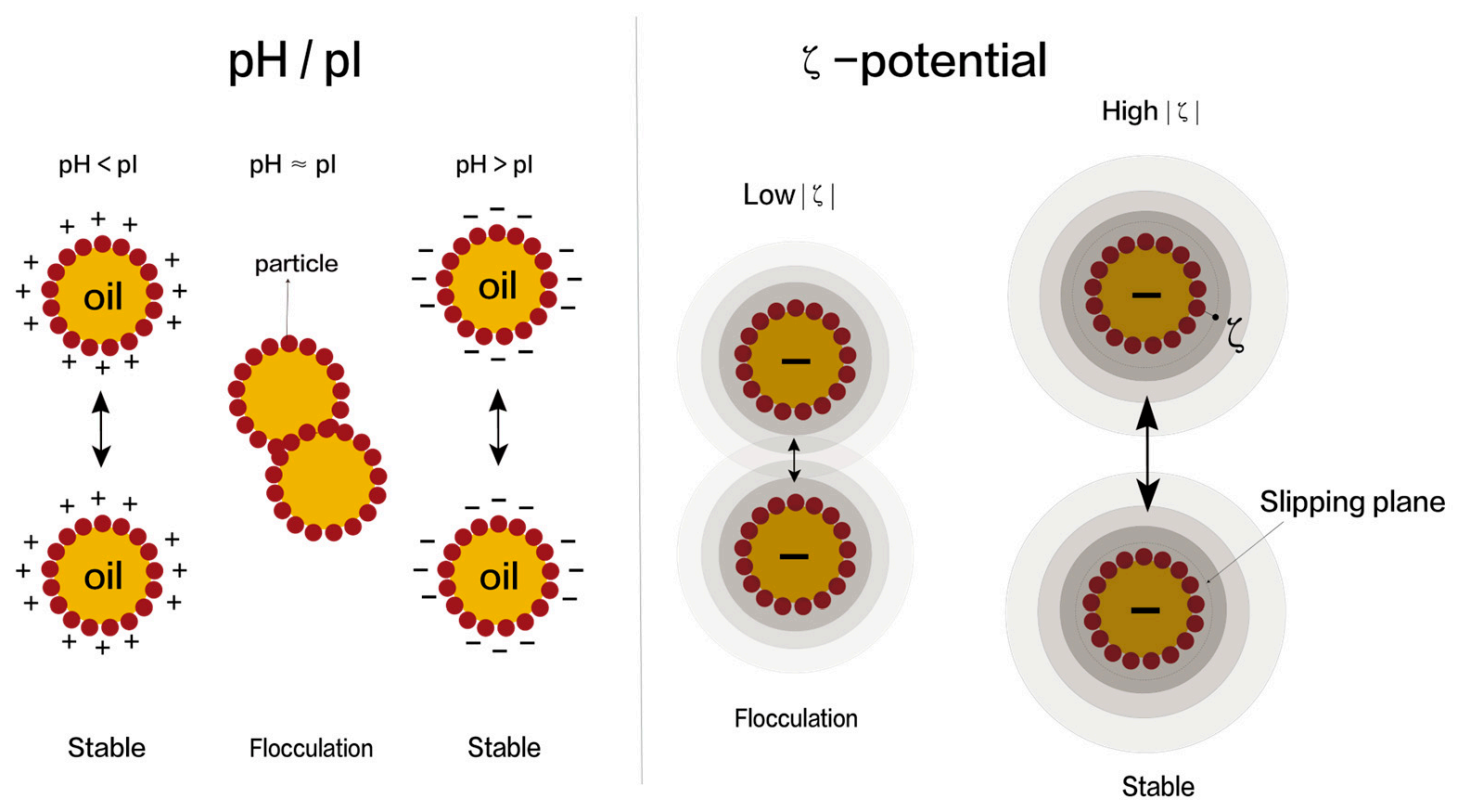
Effect of pH- and ζ-potential-mediated stabilization mechanisms in Pickering emulsions.

pH is one of the key environmental factors regulating the stability of Pickering emulsions, with particularly significant effects on pH-responsive emulsions. pH affects emulsion stability on multiple levels by altering the degree of ionization of functional groups on solid particle surfaces and the charge carried on particle surfaces. As a general rule for amphoteric particles, the surface charge state changes with pH: particles typically carry a negative charge when the pH is above their isoelectric point (pI) and a positive charge when the pH is below the pI. When pH is close to the particle isoelectric point, electrostatic repulsion between particles is extremely weak, making aggregation easy, causing significant increases in emulsion droplet size and decreased stability, or even inability to form an emulsion. Conversely, when pH is far from the isoelectric point, particles generally acquire a higher surface charge, which enhances electrostatic repulsion between droplets [34]. However, excessive surface charge may hinder rapid particle adsorption at the interface or lead to loose packing. Therefore, the relationship between surface charge and emulsion stability is not necessarily linear, and an appropriate charge level may be preferred for optimal stability. pH has an important influence on the Zeta potential of Pickering emulsions. Zeta potential is a key parameter for characterizing the electrostatic stability of colloidal dispersions. It represents the electrical potential at the slipping plane of particles or droplets and reflects their electrokinetic behavior under an applied electric field. In practice, zeta potential is commonly determined using electrophoretic light scattering (ELS). Upon application of an electric field, charged particles undergo directional electrophoretic migration, and their electrophoretic mobility is determined using laser Doppler velocimetry. The ζ-potential is then calculated from the electrophoretic mobility according to the Henry equation. The measured zeta potential is influenced by several factors: pH determines the degree of dissociation of surface functional groups and thus the net surface charge; ionic strength alters the charge-screening environment surrounding particles by compressing the electrical double layer, thereby affecting their electrophoretic mobility and zeta potential; and temperature affects the viscosity and dielectric permittivity of the dispersion medium [35,36]. The ζ-potential was calculated using the Henry equation:

(2) $$\mu_{e}=\frac{2\varepsilon_{r}\varepsilon_{0}\zeta f(\kappa a)}{3\eta }$$

where μ_e_ is the electrophoretic mobility, ε_r_ and ε_0_ are the relative permittivity of the dispersion medium and the vacuum permittivity, respectively, η is the dynamic viscosity of the dispersion medium, and f(κa) is the Henry function. Generally, a relatively high absolute value of ζ-potential indicates stronger electrostatic repulsion between droplets, thereby reducing the likelihood of droplet aggregation and coalescence and consequently enhancing the stability of the system [37]. By adjusting pH to change particle surface charge, the electrostatic interactions between emulsion droplets can be directly regulated, thereby affecting their resistance to coalescence (Figure 3). Additionally, a decrease in pH leads to higher viscosity and viscoelastic modulus, which effectively restricts the mobility of emulsion droplets. This restriction helps enhance the structural integrity and stability of emulsion gels [38].

##### Ionic Strength

**Figure 4:**
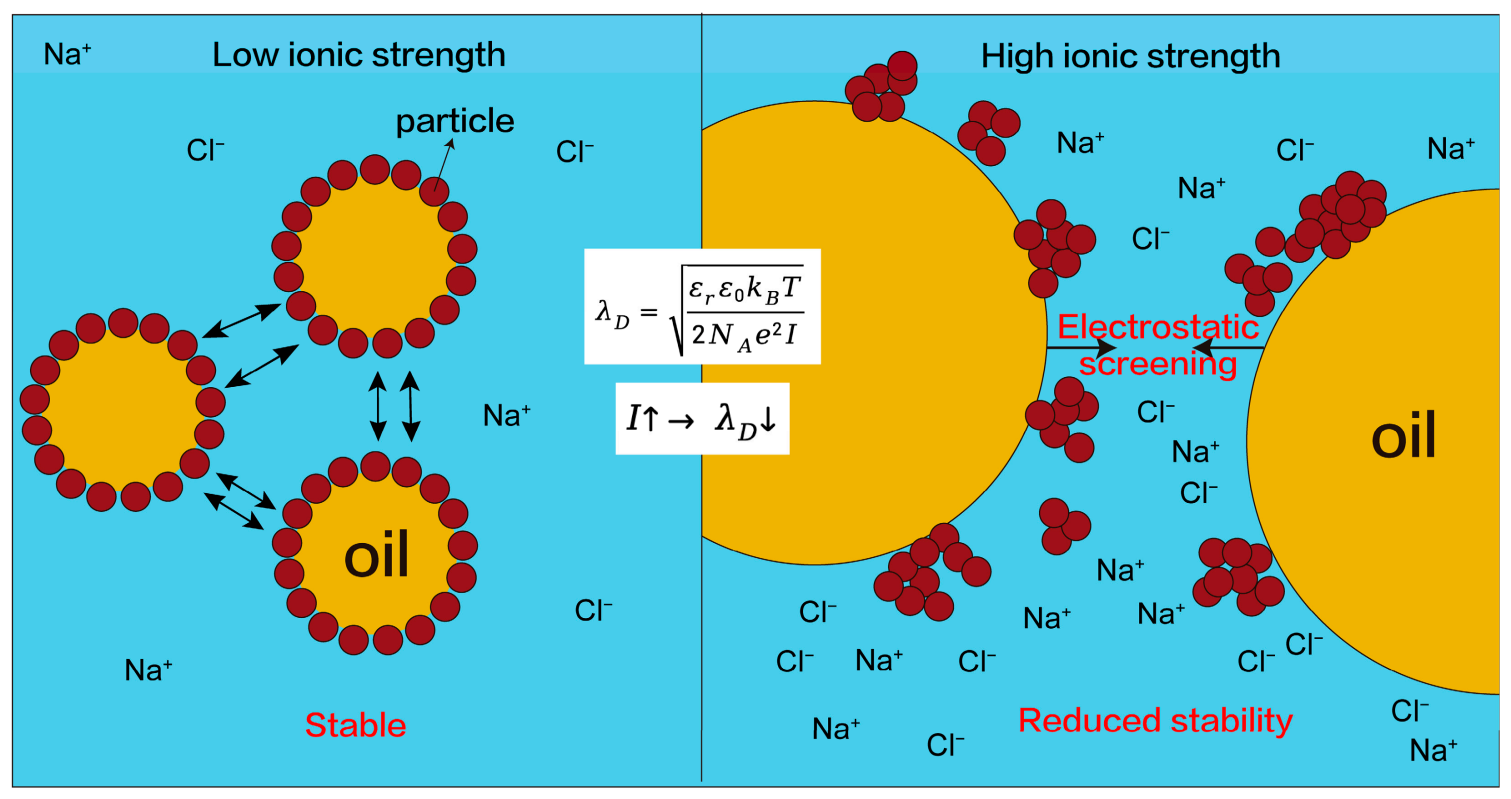
Effect of the ionic-strength-mediated stability mechanism of Pickering emulsions.

Increasing ionic strength can modulate the electrostatic repulsion between emulsion droplets, the viscoelastic modulus, and the emulsion structure, thereby affecting emulsion stability. Zhao et al., in studying the effect of ionic strength on the stability of electrostatically stabilized Pickering emulsions of silica particles and soybean hull polysaccharides, found that at low concentration ranges (0–150 mM NaCl), effective adsorption of particles at the interface and their dense packing result in a macroscopically manifested low interfacial tension. However, in this system, at high concentration ranges (150–300 mM NaCl), high ionic strength generated electrostatic shielding that greatly weakened electrostatic repulsion between particles, leading to particle aggregation and unfavorable emulsion stability. From a theoretical perspective, the Debye screening length (λ_D_)is an important parameter characterizing the effective range of electrostatic interactions within the electrical double layer in an electrolyte solution, while its reciprocal is commonly referred to as the Debye screening parameter (κ). For a symmetric monovalent electrolyte, the Debye screening length can be expressed as

(3) $$\lambda_{D}=\frac{1}{\kappa }=\sqrt{\frac{\varepsilon_{r}\varepsilon_{0}k_{B}T}{2N_{A}e^{2}I}}$$

where λ_D_ is the Debye screening length, ε_r_ and ε_0_ are the relative permittivity of the dispersion medium and the vacuum permittivity, respectively, k_B_ is the Boltzmann constant, T is the absolute temperature, N_A_ is Avogadro’s constant, e is the elementary charge, and I is the ionic strength. This relationship indicates that the Debye screening length is inversely proportional to the square root of ionic strength, meaning that increasing ionic strength shortens the characteristic range of the electrical double layer and enhances charge screening. Therefore, at higher NaCl concentrations, the electrical double layer is compressed, weakening the effective electrostatic repulsion between particles and potentially promoting particle flocculation. These flocculated particles, due to their increased size and enhanced sedimentation, are less able to effectively adsorb onto the oil–water interface, leading to insufficient interfacial coverage, enlarged droplet size, and consequently a marked decrease in emulsion stability [39,40] (Figure 4).

##### Temperature

The effect of temperature on Pickering emulsion stability is highly system-dependent, as heating may either weaken or reinforce the particle-stabilized interface depending on particle composition, concentration, and interfacial architecture [41]. Excessive heating can promote protein unfolding and aggregation, which may disrupt the integrity of the interfacial particle layer and induce droplet flocculation or coalescence. For example, walnut protein isolate nanoparticle-stabilized Pickering emulsions with a low particle concentration (0.5%) showed pronounced phase separation after boiling-water treatment, which was associated with insufficient interfacial particle coverage and heat-induced droplet aggregation [42]. Conversely, heat treatment can strengthen the interface in some protein-particle systems. Myofibrillar protein microgel-stabilized high-internal-phase Pickering emulsions remained stable after heating above 100 °C, which was attributed to enhanced interfacial hydrophobic interactions and formation of a stronger interfacial network [41].

During freezing, the mechanical stress imposed by ice crystals may disrupt the interfacial film, while freeze concentration alters the ionic strength and pH of the system, potentially weakening the electrostatic repulsion between droplets and collectively promoting droplet flocculation and emulsion destabilization [43,44]. Upon thawing, the damaged interface, resulting from particle desorption, may no longer effectively prevent irreversible droplet coalescence, ultimately leading to oiling-off and phase separation. However, the extent of freeze–thaw destabilization is also dependent on other system-specific factors, such as particle characteristics. Zhu et al. demonstrated that Pickering emulsions stabilized by unheated soy protein isolate particles exhibited substantially lower freeze–thaw stability than those stabilized by heat-treated soy protein isolate particles, suggesting that thermal pretreatment of particles can serve as an effective strategy for improving the freeze–thaw stability of Pickering emulsions [45].

## 3. Food-Grade Solid Particles for Stabilizing Pickering Emulsions

In multi-phase food systems, protein and polysaccharide particles can function as stabilizers in the form of nanoparticles or microparticles [46]. In addition to surface activity, proteins also exhibit rapid interfacial adsorption kinetics, while polysaccharide moieties can form thick steric layers that protrude into the aqueous phase, providing electrostatic repulsion against droplet aggregation [47]. However, in many cases, it is necessary to modify the surface hydrophobicity of particles or to construct protein–polysaccharide complexes. This is because using protein particles alone tends to lead to aggregation and structural instability in Pickering emulsions, while the emulsifying performance and surface activity of polysaccharide-based particles alone are relatively poor [48].

### 3.1. Protein-Based Particles

Proteins are a class of amphoteric biopolymers with good biocompatibility, widely used as emulsion stabilizers in food formulations [49]. Proteins are surface-active and can stabilize oil-in-water emulsions by forming protective films, establishing physical barriers between droplets, and enhancing emulsion stability by generating repulsive interactions (such as steric hindrance and electrostatic repulsion) between oil droplets [50]. Furthermore, the formation of protein aggregates in solution can further stabilize emulsions.

Protein-based particles used as Pickering emulsifiers mainly exist in the forms of particles, fibers, microgels, and nanogels [16]. Table 1 compiles the types, key characteristics, and main applications of protein particles commonly used in Pickering emulsions in the contemporary food industry.

The application of protein particles in Pickering emulsions offers several advantages. With respect to biocompatibility, proteins are natural biopolymers suitable for food and oral drug delivery systems. With respect to interfacial activity, proteins are amphiphilic and can effectively reduce interfacial tension. Particles of different morphologies and sizes can be obtained by adjusting preparation conditions, and they can simultaneously serve as both stabilizers and carriers for active ingredient encapsulation [17,63].

### 3.2. Polysaccharide-Based Particles

Polysaccharides are natural polymers, many of which are insoluble in both water and oil, making their particles ideal candidates for Pickering emulsion stabilization [64]. Polysaccharide-based particles have attracted extensive attention in food-grade Pickering emulsion research due to their abundant sources, good biocompatibility, biodegradability, structural diversity, tunable properties, readily modifiable surfaces, designable functionality, and strong environmental responsiveness for constructing intelligent emulsions [17,34,63,65]. The most representative polysaccharides include starch, cellulose, and chitosan/chitin. Table 2 compiles the sources, key characteristics, and main applications of polysaccharide-based particles commonly used in Pickering emulsions in the contemporary food industry.

### 3.3. Composite Particles

Proteins and polysaccharides can interact through various physical or chemical mechanisms to assemble into more efficient and stable composite structures. Such composite particles are based on electrostatic binding between proteins and polysaccharides under oppositely charged conditions—as a general rule, proteins typically carry a net negative charge above their pI and a net positive charge below it. By adjusting pH, the interaction between them can be controlled, leading to the formation of soluble complexes or even phase separation to form coacervate phases [80]. Furthermore, by controlled heating to induce protein denaturation and aggregation, or through covalent modifications such as Maillard reactions after drying and enzymatic crosslinking, the structural stability of composite particles can be further improved [47]. In addition, common composite particle types include polysaccharide–polysaccharide composite particles, protein–protein composite particles, protein–polyphenol composite particles, polysaccharide–polyphenol composite particles, ternary composite particles, and others [16]. Different particles complement and combine with each other to enhance Pickering emulsion performance. Table 3 compiles the types, key characteristics, and main applications of composite particles commonly used in Pickering emulsions in the contemporary food industry.

Compared with single-component particles, composite particles exhibit significant advantages in Pickering emulsions: synergistically enhanced stability—proteins provide excellent interfacial activity, polysaccharides provide steric hindrance and viscosity enhancement, phenolic substances provide antioxidant capacity, and multiple substances synergistically form a denser interfacial film; tunable environmental responsiveness—by controlling electrostatic interactions through pH and ionic strength, stimuli-responsive stability/destabilization of emulsions can be achieved; improved rheological properties—composite particles can form stronger three-dimensional network structures, imparting ideal gel characteristics to emulsions; multifunctional integration—simultaneous realization of emulsification, antioxidation, controlled release, and other functions; and printability—high-internal-phase emulsions stabilized by composite particles meet the rheological requirements for 3D printing and are suitable for complex food structural design [92,99,100].

## 4. Formation and Stabilization Mechanisms of Pickering Emulsion Gels

### 4.1. Formation Mechanisms

**Figure 5:**
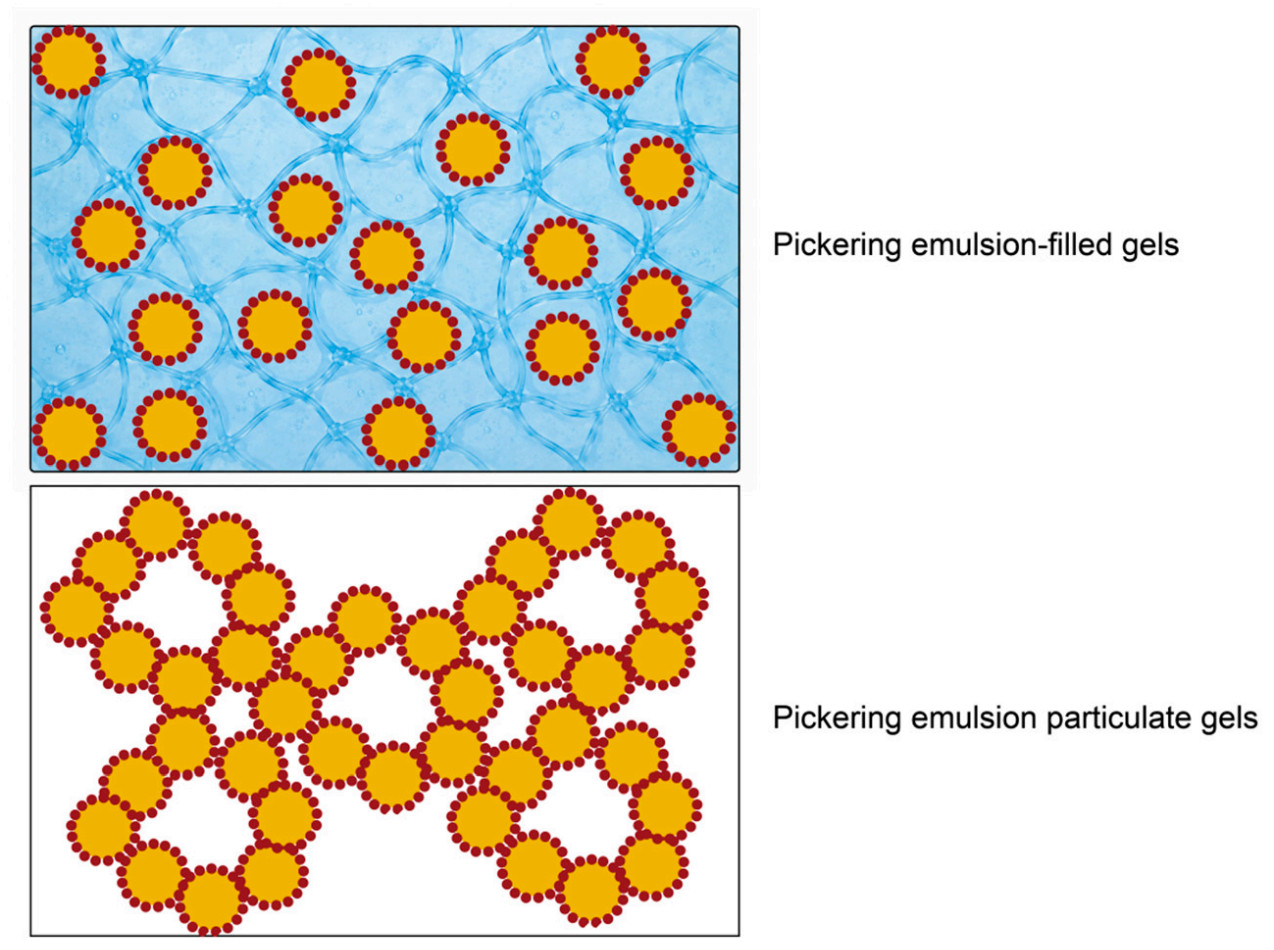
Schematic illustration of Pickering emulsion-filled gels and Pickering emulsion particulate gels.

The preparation of emulsion gels generally involves two steps: first, the preparation of the emulsion; second, gelation of the continuous phase of the emulsion by mixing with a gelling agent followed by a series of induction methods (e.g., heat treatment, enzymatic treatment, acidification, and ion addition), thereby transforming the emulsion into a solid-state gel [9]. Based on differences in gel network formation, Pickering emulsion gels can be classified into Pickering emulsion-filled gels and Pickering emulsion particulate gels (Figure 5).

Emulsion-filled gels refer to the dispersion of Pickering emulsions in a pre-existing or simultaneously formed gel matrix (such as polysaccharide or protein gels), where oil droplets serve as fillers fixed and immobilized within the gel network [101]. The gel network is typically formed through thermal, ionic, enzymatic, or acid-induced gelation. During thermal gelation, proteins unfold upon heating and expose hydrophobic groups and sulfhydryl groups, followed by aggregation and cross-linking through hydrophobic interactions, disulfide bonds, and other intermolecular interactions to form a three-dimensional gel network. Ionic gelation mainly promotes the aggregation and cross-linking of proteins or polysaccharides through charge screening or ionic bridging; for example, Ca^2+^ can cross-link alginate chains to form a gel network. Enzymatic gelation can promote network formation by catalyzing covalent cross-linking between protein molecules, typically using transglutaminase. Acid-induced gelation reduces the pH toward the isoelectric point of proteins, thereby decreasing intermolecular electrostatic repulsion and promoting protein aggregation and subsequent gel network formation [9,102]. In emulsion-filled gels, however, the droplets are not merely immobilized within the gel matrix; as dispersed fillers, their interactions with the surrounding network also contribute to the mechanical response of the final gel. For conventional emulsions, this contribution is commonly described using the Palierne emulsion model [103]:

(4) $$G_{r}=\frac{1+3H\phi }{1-2H\phi }$$

where the terms describe the modulus ratio of the composite and continuous phases, the droplet volume fraction, and the contribution of droplet and interfacial properties. However, Equation (4) was derived for conventional emulsions and does not explicitly account for the solid-particle layer characteristic of Pickering interfaces. Its application here should therefore be regarded only as a qualitative reference for understanding filler–matrix coupling, rather than as a complete constitutive description of Pickering emulsion-filled gels. In Pickering systems, adsorbed particles create a core–shell droplet structure and increase the effective dispersed-phase volume fraction; particle wettability, the ratio of droplet to particle size, and droplet aggregation or jamming can consequently alter the viscosity and rheological response [104]. The particle-coated interface can also modify creaming or sedimentation behavior because the interfacial particles change the effective size and density of the droplets, with the three-phase contact angle playing an important role [105]. Accordingly, the rheology of Pickering emulsion-filled gels reflects not only droplet volume fraction, size, and droplet–matrix mechanical coupling, but also particle-mediated interfacial contributions that are absent from the classical Palierne treatment. When strong mechanical coupling exists between the droplets and the gel matrix, the droplets can act as active fillers and contribute to stress transfer, thereby increasing the overall modulus; otherwise, they behave more like inactive fillers [101]. The coexistence of a continuous gel matrix and dispersed Pickering emulsion droplets enables flexible regulation of the rheological properties of emulsion-filled gels through modulation of both the gel network and droplet–matrix/interfacial-particle interactions. This structural tunability enables emulsion-filled gels to reproduce desirable textural and mechanical characteristics in fat-reduced foods, while droplet immobilization of droplets within the gel matrix also provides a suitable microstructure for the encapsulation and controlled release of bioactive compounds [11,106].

Emulsion particulate gels refer to systems that do not require external gelling agents but rely solely on the self-assembly, stacking, or bridging of high-concentration or strongly interacting Pickering emulsion droplets into three-dimensional network structures, commonly found in high-internal-phase Pickering emulsions [101]. As the volume fraction of the oil phase increases, the droplets gradually become crowded, come into contact with each other, and deform. They also form a continuous network through the interactions between the interfacial particle layers, while their packing and deformation directly contribute to the solid-like elasticity of the system. For conventional concentrated emulsions, this crowding-induced elasticity is commonly represented by the scaling relationship proposed by Mason et al. [107]:

G′_0_/(σ/R_d_)∼ϕ(ϕ − ϕ_c_) (5)

where the terms denote the storage modulus, interfacial tension, droplet radius, droplet volume fraction, and critical packing volume fraction (approximately 0.635), respectively. In conventional emulsions, Equation (5) relates the onset and growth of elasticity above close packing to droplet deformation and interfacial restoring stress. As with the Palierne model, however, this relationship does not explicitly incorporate a jammed or interacting layer of solid particles at the droplet interface and therefore cannot, by itself, fully describe Pickering high-internal-phase systems. A recent review of high-internal-phase-ratio emulsions and foams emphasizes that Pickering systems may differ from surfactant-stabilized emulsions because solid nanoparticles affect the effective droplet volume, interfacial mechanics, droplet–droplet interactions, yielding, viscosity, and storage modulus [108]. Thus, Equation (5) is retained as a baseline description of deformation-driven elasticity, while the actual rheology of Pickering emulsion particulate gels should additionally consider particle-layer rigidity and rearrangement, particle-mediated adhesion or bridging, aggregation/jamming, particle wettability, and particle-to-droplet size ratio [104,108]. These particle-specific effects can reinforce or otherwise modify the network response predicted from droplet crowding alone. The resulting solid-like and self-supporting properties, particularly in high-internal-phase systems, provide structural advantages for applications requiring shape retention, such as 3D food printing [109].

### 4.2. Interfacial-Network Coupled Stabilization Mechanisms

Soy protein, peanut protein, whey protein, and others have been found to be good proteins for preparing emulsion gels [110]. After a series of induction treatments, protein molecules in the continuous phase of protein-containing emulsions crosslink to form three-dimensional gel networks. These networks physically embed and immobilize oil droplets while retaining moisture, endowing the system with high storage, freeze–thaw, and oxidative stability. Brito-Oliveira et al. found that emulsion gels prepared using soy protein isolate–solid lipid particle complexes for embedding β-carotene could retain 90% of the β-carotene after 30 days of storage, attributed to the three-dimensional network structure formed [111]. The formation of three-dimensional network structures is the primary source of stability in protein emulsion gels [112].

The stability of polysaccharide emulsion gels relies mainly on interfacial adsorption, hydrogen bond crosslinking, and ionic crosslinking. Torres et al. prepared natural starch emulsion gels using modified starch and natural wheat starch, in which the hydrophobic groups of modified starch were directionally arranged on oil droplet surfaces, forming hydrophobic network structures between adjacent oil droplet starch and modified starch in the continuous phase [113]. Additionally, the hydroxyl groups on adjacent natural starch molecules can interact through hydrogen bonds, enhancing the interaction between particles and the natural starch gel matrix and strengthening emulsion gel stability.

Composite emulsion gels, with composite protein emulsion gels and protein–polysaccharide emulsion gels being the most common, rely primarily on the three-dimensional network structures formed internally for stability, while the ratio and interactions between different components further affect the conformation of this network, thereby influencing stability. For example, Sun et al. utilized the interaction between sodium alginate and composite proteins of whey protein isolate (WPI) and soy protein isolate (SPI) to promote the conversion of disulfide bonds from intramolecular to intermolecular conformations, enhancing the density and water-holding capacity of the gel network. Liu et al. found that increasing the proportion of whey protein in the matrix could enhance hydrogen bond and disulfide bond crosslinking, forming a denser gel network and substantially improving gel strength [114,115].

Pickering emulsion stability does not necessarily predict the mechanical strength or long-term structural stability of the resulting gel. Emulsion destabilization involves flocculation, coalescence and Ostwald ripening [116], whereas gel stability depends on network integrity and droplet–matrix interactions, with syneresis and fracture representing distinct failure modes [117,118]. These functions are coupled but not interchangeable: particle-covered interfaces preserve droplet integrity, whereas the network constrains droplet rearrangement. This distinction is particularly important for flocculation. In CNC/APP-stabilized Pickering high-internal-phase emulsions, higher CNC contents promoted depletion flocculation and formation of a mechanically robust gel-like network, while the particle-covered droplets retained resistance to coalescence [119]. Thus, droplet association can support network formation without necessarily indicating interfacial failure; its effect becomes detrimental when the interface can no longer prevent coalescence. Mechanical performance nevertheless cannot be inferred from droplet preservation alone. In conventional emulsion-filled gels, reducing the size of bound, nonaggregated droplets decreased fracture strain, and the magnitude depended on gelling-agent concentration [118]. Although this result does not establish the same relationship in Pickering gels, it identifies a relevant design question. Droplet size and particle-mediated droplet–matrix coupling should be considered together when interpreting fracture behavior. In addition, processing and storage conditions, such as heating and freeze–thaw cycles, can affect both the droplet interface and the gel network. Temperature variations may induce structural rearrangements of the interfacial layer and gel network, whereas ice crystal formation and water redistribution during freeze–thaw cycles may lead to water separation, droplet aggregation, and network damage [120]. Therefore, for food applications, Pickering emulsion gels should be comprehensively evaluated in terms of emulsion stability, gel network stability, thermal stability, and freeze–thaw stability to assess their long-term storage and processing stability.

## 5. Applications of Pickering Emulsion Gels in the Food Industry

### 5.1. As Solid Fat Replacers

Fat is an important flavoring substance in food, particularly in meat products. However, long-term intake of saturated fats can lead to coronary heart disease, cardiovascular disease, atherosclerosis, and other diseases [121]. Therefore, consumer concerns about the harmful effects of fat consumption and higher requirements for food safety and nutrition have collectively attracted attention to fat replacers.

**Figure 6:**
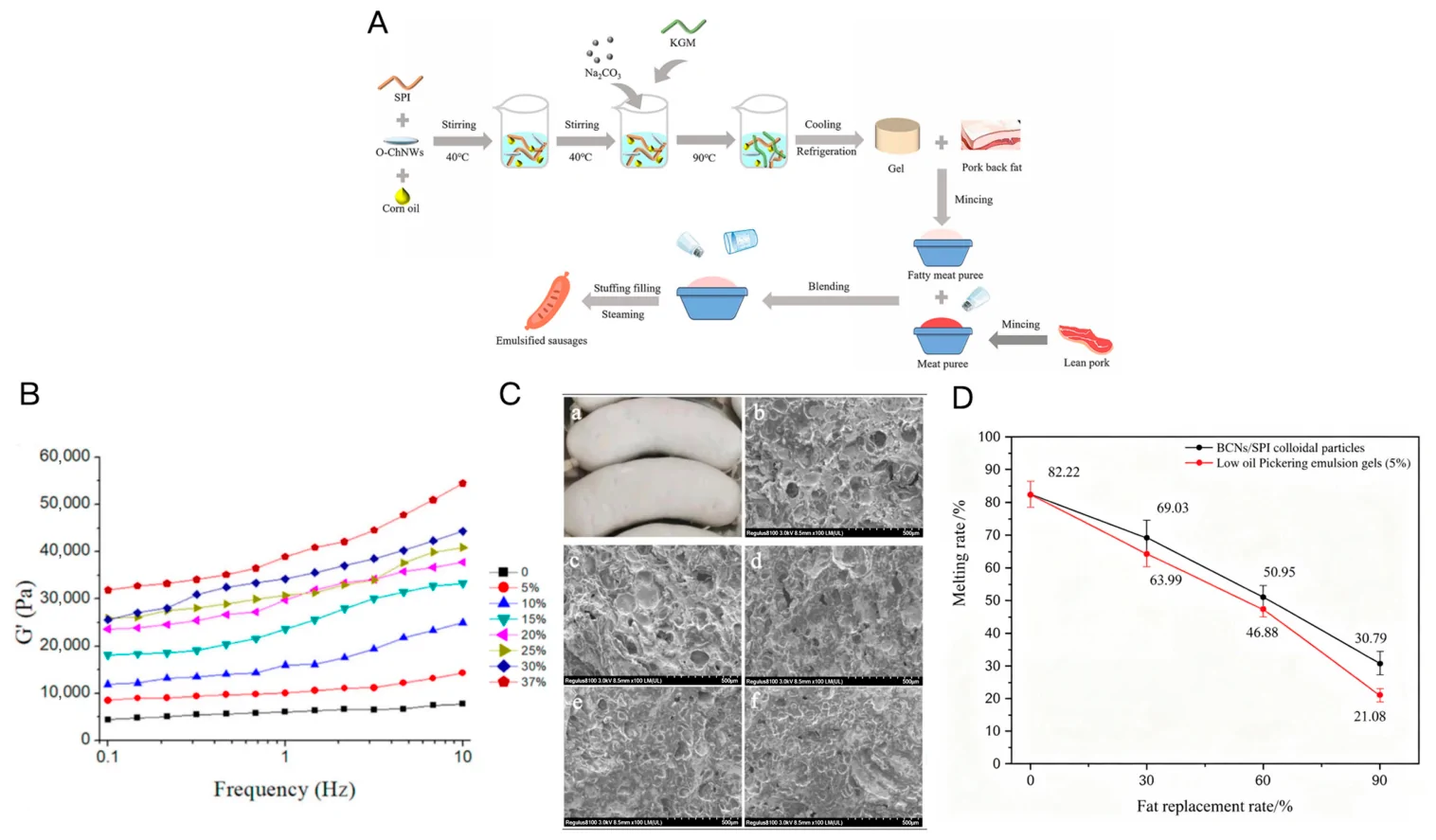
(**A**) Schematic diagram of soy protein isolate composite gel replacing pork loin fat for preparation of low-fat emulsified sausage [123]. (**B**) Influence curves of Pickering emulsion gels based on Flammulina velutipes polysaccharide nanoparticles on sausage elastic modulus at different replacement amounts [125]. (**C**) Appearance and scanning electron microscopy (SEM) images of sausages prepared with Pickering emulsion as a pork backfat substitute. (a) Appearance of the sausages. (b) SEM image of control sausages prepared with pork backfat without replacement. (c–f) SEM images of sausages with 25%, 50%, 75%, and 100% of the pork backfat replaced by Pickering emulsion, respectively, Scale bars: 500 μm [126]. (**D**) Effect curves of BCNs/SPI composite colloid or low-oil Pickering emulsion gels at different FPE replacement amounts on ice cream melting rate at 45 min [127].

The oil phase of fat-replacing Pickering emulsions (FPE) generally consists of plant oils—such as olive oil, sunflower oil, corn oil, and coconut oil—which are rich in unsaturated fatty acids and serve as effective alternatives to animal fats. The aqueous phase generally consists of water and gel network-forming agents (such as proteins, polysaccharides, inulin, and other unadsorbed components), imparting solid-like textural properties to the system [122]. Currently, Pickering emulsion gels are mainly applied in the processing of meat products such as sausages to make low-fat products. For example, as shown in Figure 6A, in a study developing soy protein isolate composite gels as fat replacers, Zhang et al. used corn oil as the oil phase and a mixed aqueous solution of soy protein isolate, konjac glucomannan, and oxidized chitin nanowhiskers as the aqueous phase. A SPI-based composite gel was prepared by homogenization and water-bath heating, which replaced pork back fat in different proportions and was combined with lean pork, salt, sugar, and other ingredients to produce low-fat emulsified sausages [123,124].

FPE possesses unique rheological properties and performance similar to fat, along with the advantages of non-toxicity, high tunability, and high stability, demonstrating great potential as a fat replacer. First, in terms of rheological properties, Pickering emulsion gels utilize the “filling effect” of their interfacial particles to significantly enhance the elasticity and cohesion of the system, thereby precisely simulating the mechanical response of animal fat. As shown in Figure 6B, Yang et al. demonstrated that Pickering emulsion gels based on Flammulina velutipes polysaccharide nanoparticles could fill the three-dimensional network matrix of sausages, simulating the filling effect of fat. As the substitution amount increased, the product’s elastic modulus also increased, making its rheological properties increasingly similar to those of products containing animal fat [125]. Second, in terms of microstructural similarity, as shown in Figure 6C, research has demonstrated that as the FPE substitution amount increases, the three-dimensional network structure in the sausage matrix becomes denser, and this structural uniformity effectively mimics the supporting role of natural fat crystals in the food skeleton [126]. Some additives can also regulate the texture of fat replacers; for example, konjac glucomannan (KGM) has been effectively used to replace pork back fat in dry-fermented sausages, and compared with the control group, 50% fat replacers improved system hardness, chewiness, and sensory acceptance [128].

Beyond the above advantages, FPE also has numerous other benefits. First, emulsion gels have a fine, elastic, soft-solid texture and three-dimensional network structure that can mimic the mouthfeel of solid animal fats while also protecting flavors and pigments [9]. Second, fat levels can be reduced, especially saturated fatty acid content. Pagliarini et al. constructed emulsion-filled gels using soy protein isolate, inulin, and soybean oil as animal fat replacers in Bologna sausages, and the fat content of the reformulated product was reduced by 11% to 34% [129]. Similarly, Pintado et al. replaced fat in Frankfurt sausages with emulsion gels prepared from chia seed flour and olive oil, reducing fat content by up to 40% [130]. Additionally, Pickering emulsion gels can reduce oxidative susceptibility. Freire et al. prepared refrigerated gels by combining sodium caseinate, WPI, and soy protein with a natural concentrated tannin extract, finding that these gels had low sensitivity to oxidation, making them suitable as fat replacers [131]. Finally, FPE can also improve the melt resistance of low-fat ice cream. As shown in Figure 6D, in a 45 min melting experiment, the melting rate of ice cream decreased with increasing substitution of the low-oil Pickering emulsion gels stabilized by bacterial cellulose nanofibers/soy protein isolate (BCNs/SPI). When the fat replacers rate reached 90%, the melting rate of ice cream was the lowest at only 21.08% [127].

Currently, FPE research mainly focuses on reproducing the structural and rheological properties of animal fat; however, consumer acceptance is also influenced by multiple sensory attributes [132]. In terms of texture and overall sensory quality, Qin et al. regulated the particle structure of pea protein microgels by adjusting their preparation conditions and found that Pickering emulsion gels prepared with microgels of appropriate structural properties produced favorable sensory performance when used as fat replacers in ice cream. However, higher replacement levels resulted in pronounced ice-crystal perception and beany odor, indicating that particle structure and formulation should be optimized according to the specific food matrix [133]. In terms of juiciness, Yan et al. found that replacing pork fat with high-internal-phase Pickering emulsions stabilized by microwave-treated PPI–GG composite particles increased immobilized water and decreased free water as the replacement level increased, thereby improving water retention and juiciness, with the highest juiciness score observed at a 75% replacement level. In contrast, the flavor score decreased with further increases in the replacement level, which was attributed by the authors to the reduced contribution of characteristic flavor precursors from animal fat [134]. Therefore, rather than simply increasing the replacement level, optimizing particle composition and emulsion structure to improve water retention while minimizing the loss of characteristic product flavor may be more conducive to achieving higher consumer acceptance.

### 5.2. Delivery Systems for Bioactive Substances

Bioactive substances (such as vitamins, carotenoids, polyphenols, and essential oils) possess antibacterial, anti-cancer, anti-inflammatory, and antioxidant properties, but their practical applications are limited by factors such as bioavailability, chemical stability, and water solubility [135]. Pickering emulsion gels, with their high loading capacity, exceptional stability, and tunable performance, can isolate active substances from oxygen, light, and heat, making them ideal delivery carriers [136].

#### 5.2.1. Encapsulation Methods

**Figure 7:**
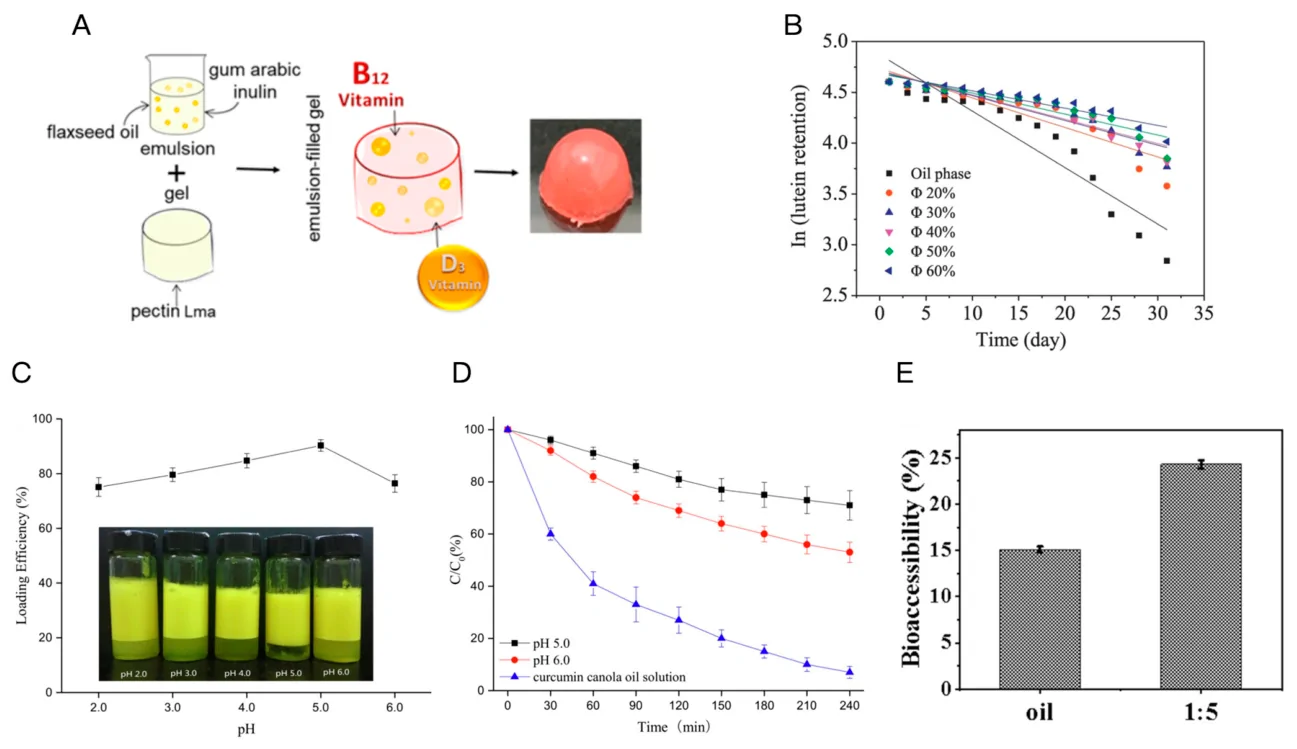
(**A**) Schematic diagram of co-encapsulation of vitamins D3 and B12 in inulin–gum arabic emulsion gel [138]. (**B**) Storage stability of lutein-loaded oil phase and Pickering emulsions stabilized by octenylsuccinate quinoa starch (OSQS) particles as a function of oil phase volume fraction Φ [139]. (**C**,**D**) Loading efficiency of curcumin in Pickering emulsions and emulsion gels at different pH (**C**); time-course photostability curves of curcumin in Pickering emulsions and emulsion gels at different pH conditions (**D**) [140]. (**E**) Comparison of bioaccessibility of astaxanthin in oil and in G/SNPs 1:5-stabilized Pickering emulsion gels [141].

Pickering emulsion gels utilize their multiphase structures to achieve compartmentalized encapsulation of active substances, with encapsulation methods mainly including oil-phase encapsulation, aqueous-phase gel network encapsulation, and composite system encapsulation. The selection of encapsulation methods is primarily determined by the hydrophilicity or hydrophobicity of the bioactive compounds, as well as their desired release site and release behavior. Oil-phase encapsulation is the most common in O/W emulsion systems, suitable for lipid-soluble active substances (curcumin, β-carotene, lutein, astaxanthin, VD3, essential oils, etc., Figure 7). In this case, the bioactive compounds are mainly located within the oil droplets, while the interfacial particle layer serves as a physical barrier that minimizes their direct contact with the external aqueous phase, oxygen, and other environmental factors, thereby achieving both encapsulation and protection. Additionally, the enzymatic stability of the interfacial particles can further modulate the exposure of oil droplets and the rate of lipid hydrolysis during gastrointestinal digestion, thereby affecting the release of lipophilic bioactive compounds. For example, particles resistant to enzymatic hydrolysis (silica, cellulose, chitosan) adsorbed on oil droplet surfaces can effectively delay or inhibit lipid digestion, thereby achieving regulation of lipid hydrolysis and/or controlled release of lipophilic active components [137].

Unlike lipid-soluble active substances, hydrophilic active substances (water-soluble vitamins, polyphenols, minerals, etc.) are encapsulated in the inner aqueous phase or other hydrophilic compartments. For W/O/W Pickering double emulsions, hydrophilic active substances are sequestered in the internal aqueous phase, with the external aqueous phase and interfacial barriers limiting their diffusion and untimely release. Maeda et al. utilized the synergistic action of cellulose nanocrystals, sodium alginate, and lecithin to construct a stable Pickering double emulsion (W/O/W), successfully encapsulating the hydrophilic vitamin B3 ionic liquid in the inner aqueous phase with an encapsulation efficiency of up to 98.65% and achieving good controlled-release effects [142].

The presence of different phases in double emulsion systems makes composite systems (simultaneous oil phase + aqueous phase encapsulation of hydrophobic and hydrophilic functional factors) possible. As shown in Figure 7A, Ghiraldi et al. developed an emulsion gel-based confectionery using inulin, pectin, and gum arabic to protect and deliver vitamins B12 and D3 in the aqueous and lipid phases, respectively. There was no difference in active vitamins between the day of manufacture and after 30 days of storage, indicating that the gel showed great potential in limiting exposure to light and oxygen [138]. The greatest advantage of composite systems is the simultaneous delivery of functional factors with synergistic functions. In 2015, Aditya et al. first co-encapsulated hydrophilic catechin and hydrophobic curcumin in a W/O/W double emulsion gel system, and found that stability in simulated gastrointestinal fluids was significantly improved, with bioaccessibility 4 times higher than that of unencapsulated curcumin and catechin [143].

#### 5.2.2. Delivery Advantage Mechanisms

Pickering emulsion gels provide protective effects on bioactive substances. First is the interfacial particle barrier and environmental isolation. As shown in Figure 7B, Li et al. prepared OSQS particle-based emulsion gels as carriers for lutein and found that the retention index of lutein in emulsion gels could reach up to 55.38% after 31 days of storage, significantly better than the pure oil phase system (only 17.51%). This is related to the dense packing layer at the oil/water interface [139]. Second is light/heat protection. As shown in Figure 7C,D, Lv et al. fabricated Pickering emulsion gels stabilized by food-grade WPI gel particles, and the Pickering emulsion gels containing curcumin exhibited excellent loading efficiency and photodegradation stability [140]. Emulsion gels prepared from nanoparticles stabilized by soy β-conglycinin and polyphenol complexes can be used for encapsulating β-carotene to protect it from high temperature and oxidation, thereby extending shelf life [144].

Upon gastrointestinal digestion, the interfacial particle layer, droplet microstructure and gel network of Pickering emulsion gels undergo gradual structural changes that affect lipid digestion and bioactive release. In the gastric phase, pepsin hydrolyzes protein-based interfacial particles or gel networks, compromising interfacial film integrity. In the intestinal phase, bile salts and pancreatic lipase act on the oil–water interface and triglycerides, promoting lipolysis and mixed micelle formation, through which released bioactives are transported to intestinal epithelial cells for absorption. The particle composition, size, charge, and interfacial structure govern the rate of lipid hydrolysis by modulating interfacial stability and enzyme accessibility to oil droplets [137]. Thus, Pickering emulsion gels require a delicate balance between structural protection and triggered release to achieve efficient bioactive delivery.

Pickering emulsion gels have the potential to enhance both the bioaccessibility and bioavailability of bioactive compounds. In vitro digestion (Figure 7E) showed that the bioaccessibility of astaxanthin in Pickering emulsion gels stabilized by gliadin/starch nanocomposites (1:5) was 1.5 times that in oil [141]. Bioavailability is also affected by gel strength; Hao et al. found that reducing Pickering emulsion gel strength could lead to a significant decrease in the bioaccessibility of β-carotene, possibly due to partial degradation of β-carotene by gastric acid. The study also showed that increasing β-carotene loading promotes lipid decomposition and mixed micelle formation, thereby improving its bioavailability [145].

### 5.3. Applications in 3D-Printed Foods

#### 5.3.1. Advantages of Pickering Emulsion Gels in 3D Food Printing

**Figure 8:**
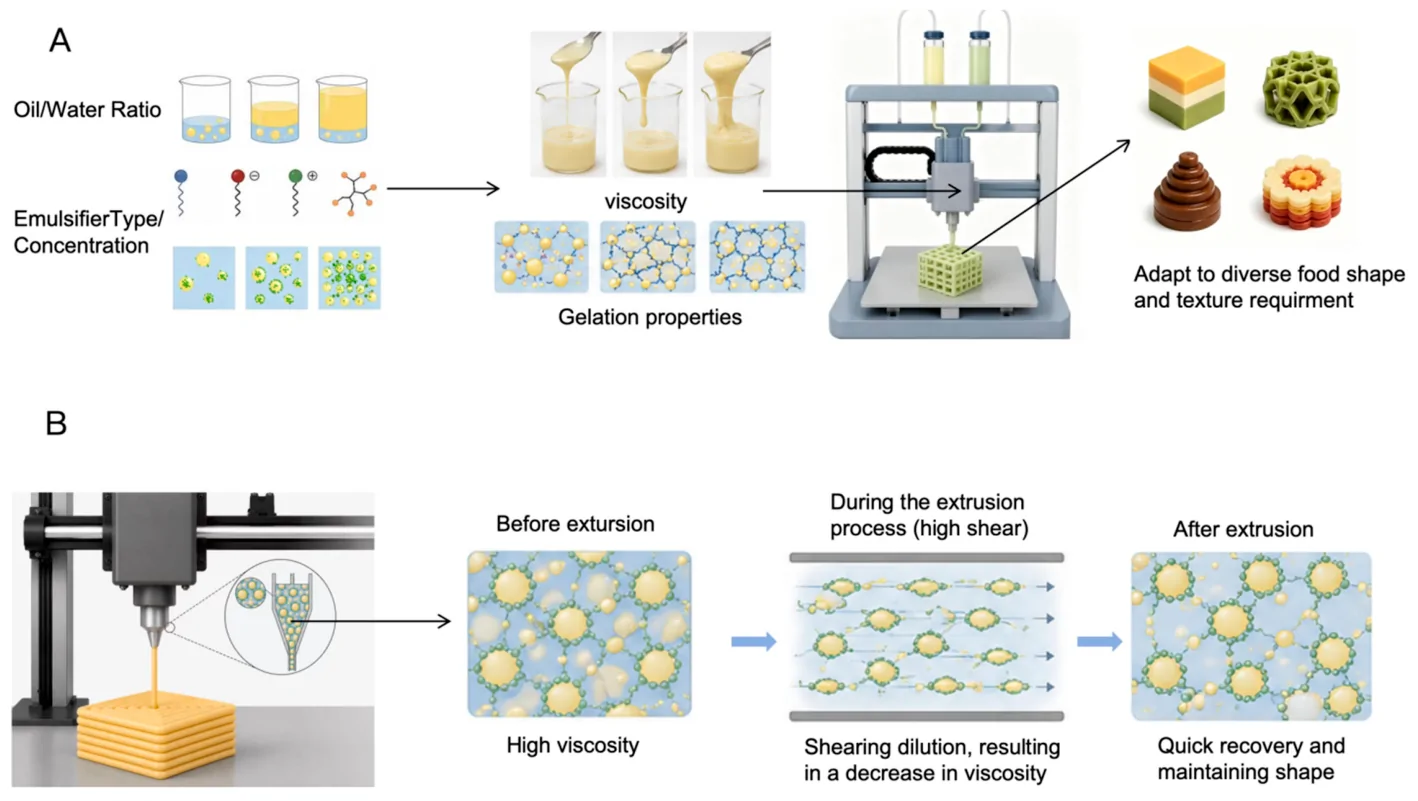
(**A**) Tunable factors of Pickering emulsion gels rheological properties and their effects on 3D-printed food shape and texture adaptability. (**B**) Schematic diagram of shear-thinning behavior and shape recovery of Pickering emulsion gels during 3D printing extrusion. The arrows in the middle schematic indicate the direction of shear-induced flow during extrusion.

In recent years, 3D printing technology has attracted considerable attention in the food sector, as it enables the personalized customization of food shape and structure through digital design. Pickering emulsion gels exhibit advantages such as tunable rheological properties and structural adaptability, making them promising materials for food 3D printing [146]. First, the rheological properties of Pickering emulsion gels can be regulated through formulation factors such as the oil-to-water ratio and particle type and concentration, thereby modulating the viscosity, storage modulus, and gel properties of the system to meet the printing requirements of different food structures (Figure 8A) [147]. Second, Pickering emulsion gels typically exhibit pronounced shear-thinning properties, resulting in reduced viscosity during nozzle extrusion and consequently lower extrusion resistance. After cessation of shear, partial recovery of the particle network and droplet structure can rapidly restore the structural strength of the deposited material, thereby facilitating the continuity and shape fidelity of the printed filaments (Figure 8B) [148]. Additionally, higher yield stress and stronger gel networks can enhance resistance to deformation after deposition, allowing printed structures to withstand their own weight and the stresses imposed by subsequently deposited layers, thereby improving self-supporting capability. For example, when fucoidan was incorporated as ink for 3D printing in a pectin–salmon by-product protein composite Pickering emulsion, the final product demonstrated high self-supporting capability and graphical precision [149].

#### 5.3.2. Printing Performance Regulation Strategies

The 3D printing performance of Pickering emulsion gels is influenced by multiple factors, and through rational regulation strategies, their rheological properties, self-supporting capability, and printing precision can be optimized.

Protein particle concentration directly determines the structural support capacity of Pickering emulsion gels. Wu et al. found that increasing the protein particle concentration increased the viscosity and storage modulus of the system, thereby improving the accuracy and stability of the printed structures, which reached 99.27% and 99.72%, respectively, at a protein particle concentration of 1.6% [150]. Wu et al. similarly observed that increasing the concentration of SPI microgel particles enhanced the shape retention of the printed structures. This improvement can be attributed to the formation of a denser particle network and enhanced structural support between droplets at higher particle concentrations, making the deposited filaments less susceptible to deformation [151]. However, excessively high particle content increases extrusion resistance and reduces extrudability [152]. From the general mechanism of extrusion-based 3D food printing, excessive flow resistance may further lead to insufficient extrusion, discontinuous material deposition, or filament breakage, thereby reducing printing accuracy and structural integrity [153]. Therefore, a balance must be struck between extrudability and structural support.

The oil phase fraction affects printing performance by altering the dispersed-phase volume fraction, droplet packing, and interfacial particle coverage, which in turn regulate the viscosity, storage modulus, and structural strength of the system. Wu et al. found that, within a certain range, increasing the oil phase fraction increased the viscosity, storage modulus (G′), and gel strength, suggesting that a higher internal-phase volume fraction promoted tighter droplet packing and enhanced structural support between droplets, thereby improving printing resolution, shape fidelity, and post-printing structural stability. However, when the oil content increased to 30%, the excessively high viscosity and hardness may have hindered extrusion, resulting in greater printing deviations and lower accuracy [150]. Li et al. also reported that an excessively high oil phase fraction may generate additional oil–water interfaces that cannot be sufficiently covered by the limited number of particles, resulting in increased droplet size and reduced emulsion stability. This may lead to filament breakage during extrusion and insufficient interlayer adhesion [152]. Therefore, an appropriate oil phase fraction can improve shape fidelity and post-printing structural stability by promoting favorable droplet packing and structural support, whereas excessive oil contents may compromise printing resolution due to reduced interfacial stability or excessive thickening of the system.

The incorporation of polysaccharides can improve the printing performance of Pickering emulsion gels by strengthening the continuous-phase network. On the one hand, polysaccharides can increase the viscosity of the aqueous phase and restrict droplet movement; on the other hand, they can interact with protein particles through hydrogen bonding, hydrophobic interactions, or electrostatic interactions, thereby forming or reinforcing the structural network in the continuous phase and enhancing structural support and resistance to deformation. Zheng et al. reported that increasing the xanthan gum concentration enhanced shear-thinning behavior, thermal stability, and structural recovery, thereby improving printing resolution and structural fidelity [154]. Wei et al. further demonstrated that the effects of different polysaccharides varied substantially; for example, the chitosan-containing system exhibited a printing deformation rate below 5.5%, while different polysaccharides exerted distinct effects on shape retention by modifying protein–polysaccharide interactions and network structures [155]. However, insufficient polysaccharide concentrations may fail to provide adequate structural support, whereas excessive addition may result in excessive viscosity and hinder extrusion. Therefore, polysaccharide concentration should be optimized to achieve a balance between structural support and extrudability [154].

System pH is an important factor governing the structure of protein particle-stabilized Pickering emulsion gels and their 3D printing performance. Changes in pH can alter the charge states of protein particles and polysaccharides, thereby modulating electrostatic interactions between the protein particles and polysaccharides, as well as the resulting composite structures and gel networks, ultimately affecting printing resolution, shape fidelity, and post-printing structural stability. Wang et al. found that different interactions between protein particles and polysaccharides at pH 3 and pH 7 resulted in distinct microstructures in pea protein isolate-stabilized Pickering emulsion gels. The system at pH 7 formed a more uniform honeycomb-like structure and produced printed structures with precise shapes and smooth surfaces [156]. Wei et al. further demonstrated that pH directly affected structural recovery and 3D printing accuracy. The xanthan gum-containing system at pH 7 exhibited the highest structural recovery rate (97.45%), whereas the pH 3 system showed a weaker gel structure and a larger printing dimensional deviation (27.57%) [155]. Therefore, an inappropriate pH may disrupt intermolecular interactions and weaken the network structure, ultimately leading to deformation of the printed structures.

#### 5.3.3. 4D Printing

**Figure 9:**
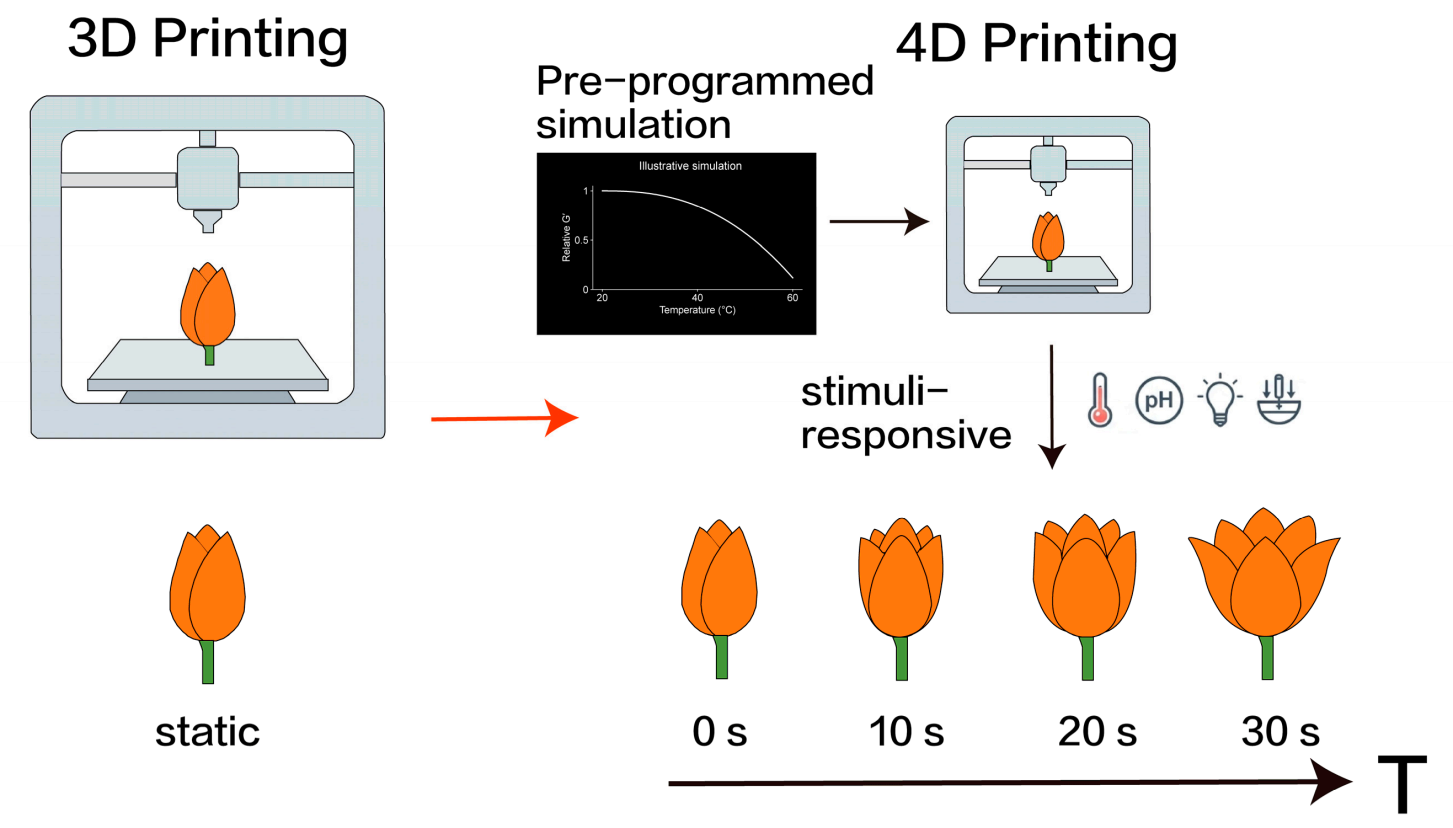
Schematic diagram of 4D printing principles.

Emerging 4D printing has attracted widespread attention in recent years. Unlike conventional stimulus-responsive 3D printing, which primarily emphasizes the response of printed materials to external stimuli such as temperature, pH, light, or moisture, 4D printing further requires such responses to be deliberately programmed through material composition, internal structure, or printing geometry, enabling the printed structures to undergo predictable, controllable, and time-dependent transformations in shape or function upon exposure to specific stimuli (Figure 9) [76,157]. Currently, 4D food printing has demonstrated diverse stimulus-triggered dynamic responses. Liu et al. achieved predictable shape transformation by predesigning the internal configuration of starch-based printed structures and exploiting differential shrinkage during dehydration [158]. Cen et al. developed a curcumin-based 4D-printable Pickering emulsion stabilized by citrus pectin–β-cyclodextrin complex particles. By preconstructing a temperature–pH-responsive system, heating-induced decomposition of NaHCO_3_ increased the pH and consequently induced a color transition of curcumin from yellow to red, enabling a predictable post-printing color change [159]. 4D printing is not limited to changes in external shape and color; new advances have also been made in internal structural transformations. Shahbazi et al. constructed Pickering high-internal-phase emulsions using hydroxybutyl-modified starch nanoparticles as stabilizers. The LCST-type thermoresponsive behavior of the hydroxybutyl chains near body temperature (~37 °C), characterized by conformational contraction driven by hydrophobic aggregation, induced shrinkage of the scaffold pores, thereby enabling temperature-triggered changes in the internal structure [160].

#### 5.3.4. Consumer Applicability

At present, studies that directly investigate the consumer acceptance of Pickering emulsion gel 3D-printed food are still relatively rare. Existing research mainly focuses on sensory characteristics and the applicability of the food for specific groups. For instance, Shahbazi et al. found that the composition of the Pickering emulsion can further influence the lubricity, creaminess, and oral coating sensation of the fat-reduced cheese analogue by regulating the printing structure and oral friction characteristics, indicating that 3D printing performance is extending from simple structural formation to consumer-perceivable food quality design [161]. Further, the structural modularity of the Pickering emulsion gels provides the possibility for personalized texture and novel food design, such as by spatially regulating the distribution of the emulsion gel in plant-based meat to improve juiciness and meat-like texture [162]. At the same time, studies targeting individuals with swallowing difficulties show that HIPEs not only meet the rheological and structural stability requirements of 3D printing but also enable the products to reach specific International Dysphagia Diet Standardisation Initiative (IDDSI) texture grades, thus balancing formation, swallowing safety, and eating quality [163]. Therefore, Pickering emulsion gel 3D printing is evolving toward a consumer-oriented platform. Future efforts should prioritize linking printing performance to sensory outcomes and population-specific needs, validated through real consumer testing.

### 5.4. Storage and Preservation

**Figure 10:**
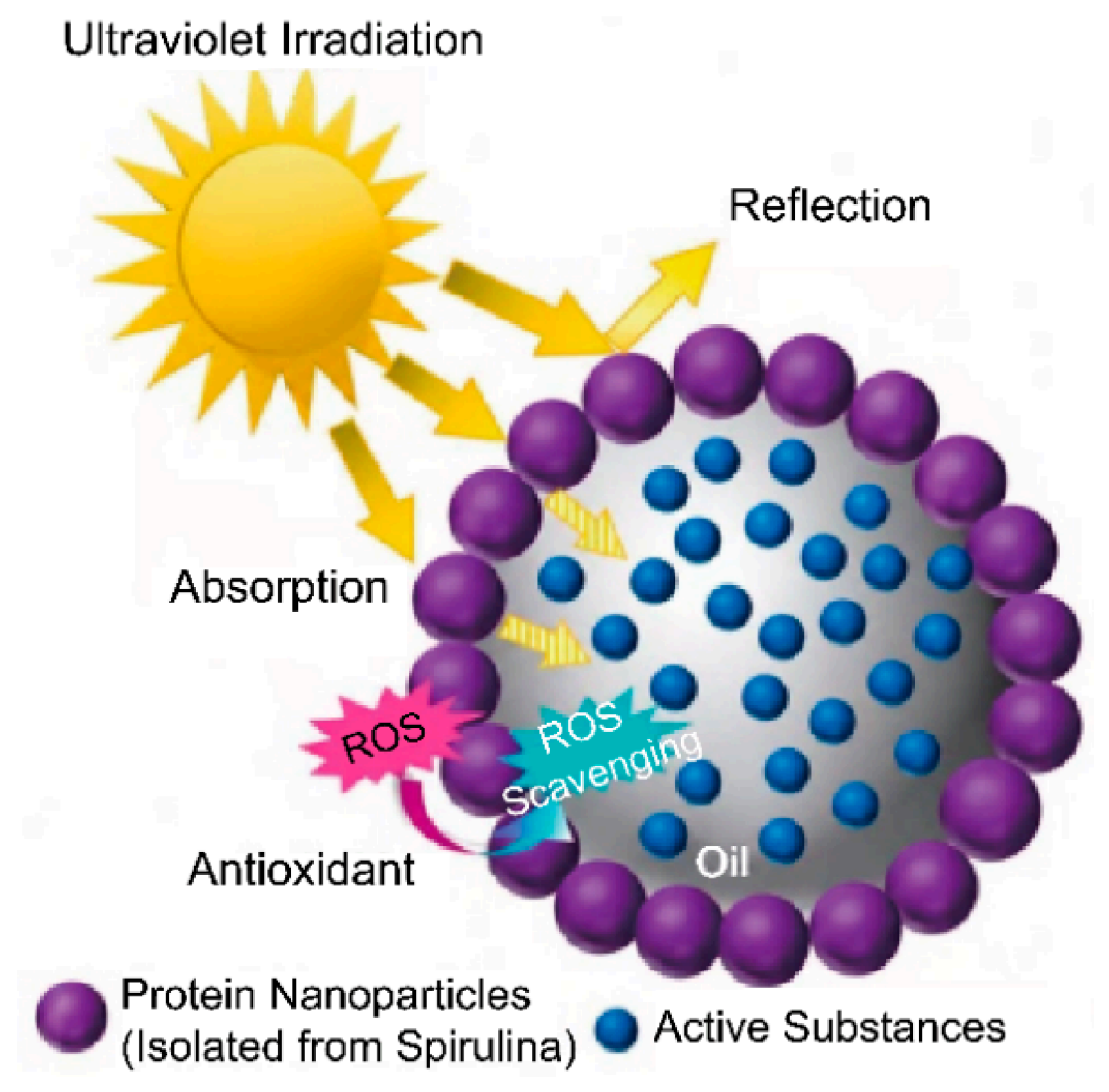
Schematic diagram of the UV protection mechanism of Spirulina protein nanoparticle-stabilized Pickering emulsion gels.

Food packaging is used to protect food from environmental factors such as ultraviolet radiation, oxygen, and pressure, reducing the harm of microbial contamination. Compared with traditional packaging, active food packaging based on Pickering emulsion gels has stronger packaging barrier properties [164]. First, the dense layer formed by solid particles at the oil–water interface can act as a physical barrier that reduces the influence of external environmental factors. High-internal-phase Pickering emulsions stabilized by Spirulina protein nanoparticles have been demonstrated to maintain excellent storage stability under UV irradiation. Research has shown that, as illustrated in Figure 10, Spirulina protein nanoparticles can generate a physical barrier at the oil–water interface of emulsions, thereby providing excellent UV protection for numerous photosensitive compounds (such as β-carotene, vitamin E, and limonene), maintaining their bioactivity [165]. Furthermore, the particles or encapsulated active substances (such as essential oils) can also provide protective functions. Tetramethylpiperidine oxide (TEMPO)-oxidized chitin nanocrystals, combined with oregano essential oil, were used to stabilize Pickering emulsion gels, which were then incorporated into a konjac glucomannan matrix by solvent casting evaporation technology to prepare active food packaging films with excellent light barrier, antioxidant, and antimicrobial properties, The incorporation of 20% emulsion reduced the water vapor permeability (WVP) of the KGM film from 9.51 to 8.71 g·mm/(m^2^·day·kPa). In terms of light barrier properties, the UV–Vis transmittance of the KGM film decreased from 75.21% to 10.96% upon the addition of 40% emulsion [166]. Furthermore, the emulsion addition level in the film-forming matrix also influences the barrier properties of active packaging. Farokhi et al. incorporated Pickering emulsions into a gelatin matrix at volume ratios of 10%, 20%, and 30%. At 10% emulsion addition, the WVP decreased from 9.25 × 10^−7^ to 8.39 × 10^−7^ g·mm/(m^2^·s·Pa), and the OP (Oxygen Permeability) decreased from 42.51 × 10^−9^ to 36.06 × 10^−9^ L·m/(m^2^·s·Pa). However, when the emulsion addition level increased to 30%, the OP rebounded to 41.75 × 10^−9^ L·m/(m^2^·s·Pa), indicating that the improvement in barrier properties is not linear and that an optimal addition level exists [167]. Beyond these functional properties, for food-contact applications, the safety of Pickering emulsion gel-based packaging should also be evaluated by assessing the migration of particles, active compounds, other formulation components, and degradation products into food under relevant storage and use conditions [168].

## 6. Challenges in the Industrial Translation of Pickering Emulsion Gels

### 6.1. Industrial-Scale Production

Scale-up production is one of the key challenges hindering the industrial application of Pickering emulsion gels. Most studies to date have been conducted at the laboratory scale, while changes in mixing and shear fields during scale-up may affect droplet breakup, particle size distribution, and interfacial structure, thereby altering the rheological properties and batch-to-batch consistency of Pickering emulsion gels. This challenge is particularly pronounced for high-internal-phase systems because their high viscosity and particle suspension requirements further complicate process scale-up [169]. In addition, conventional batch high-shear emulsification is associated with high energy consumption, heat release, and difficulties in industrial scale-up. In contrast, continuous emulsification, which can be scaled by increasing the number of identical units rather than simply enlarging a single device, together with efficient energy utilization and improved temperature control, provides a potential route toward enhanced process efficiency and large-scale Pickering emulsion production. Marques et al. employed a NETmix continuous reactor to produce Pickering emulsions continuously, demonstrating the scalability potential of this approach. However, differences in emulsion stability between continuous and batch production indicate that further optimization of process parameters is required to balance production efficiency with product consistency [170]. From a commercialization perspective, successful scale-up requires a balance between raw-material and processing costs, product consistency, and the added value of the final product. Therefore, developing cost-effective and scalable production strategies without compromising product functionality will be an important direction for the commercial translation of Pickering emulsion gels.

### 6.2. Economic Feasibility

The economic feasibility of Pickering emulsion gels depends primarily on raw material costs, processing requirements, production efficiency, and the added value generated by the resulting products. The extraction, purification, and functionalization of food-grade particles may increase material costs; therefore, developing particles that are widely available, cost-effective, and amenable to large-scale production is an important prerequisite for industrialization. Cellulose and its derivatives, for example, are derived from abundant sources and have been used to construct Pickering emulsions with good stability and food-processing applicability, highlighting their potential as cost-effective food-grade particles [171]. Meanwhile, emulsification processes such as high-pressure homogenization, high-shear homogenization, and ultrasonication may require substantial energy input and capital investment. Large-scale production also requires increased production throughput to reduce the unit processing cost, which is particularly challenging for 3D-printed foods because their industrialization is constrained by low production throughput as well as relatively high initial investment and operating costs [172]. By contrast, high-value applications such as bioactive delivery and active packaging may provide greater economic incentives if Pickering systems can improve active-compound stability, extend food shelf life, or reduce food losses, although whether these benefits can offset the additional processing costs remains to be established [173]. Therefore, systematic cost–benefit analyses should be conducted for different applications to determine the economic boundaries for the large-scale implementation of Pickering emulsion gels.

### 6.3. Regulatory Approval

Regulatory approval and safety assessment represent another important challenge for the industrialization of Pickering emulsion gels. For food-grade nano- or micron-scale particles, both the U.S. Food and Drug Administration (FDA) and the European Food Safety Authority (EFSA) emphasize the need for case-specific safety assessment considering particle size, composition, surface properties, dissolution/degradation behavior, and potential consumer exposure. The established safety history or GRAS status of a conventional material cannot simply be extended to its nanoscale form [174,175]. For example, the regulatory status of different nanocellulose materials under GRAS is not necessarily equivalent, indicating that their safety and regulatory acceptance should be evaluated according to the specific material and intended use [176]. In addition, gastrointestinal digestion and degradation, potential systemic exposure, and changes in biological properties caused by physical or chemical functionalization should also be considered in safety assessments [174,175]. For Pickering systems used for bioactive delivery, the exposure and bioavailability of the active compounds should also be considered [173]. Accordingly, within existing regulatory frameworks, safety assessment approaches specific to Pickering emulsion gels should be further refined, with particular attention to particle characteristics, GRAS status and other regulatory requirements, gastrointestinal fate, potential exposure, and the safety of associated bioactive compounds.

### 6.4. Shelf-Life Validation

Shelf-life validation is an important step in translating Pickering emulsion gels from laboratory-scale research to practical food applications. During storage, droplet aggregation, phase separation, and changes in the gel network structure may all lead to deterioration in product quality. For example, in a Pickering emulsion stabilized by canola protein microgels and developed as an animal-fat replacer, phase separation, droplet size, and rheological properties were monitored during 40 d of storage at 4 °C to evaluate storage stability [177]. For Pickering emulsion gels containing high levels of unsaturated fatty acids, lipid oxidation should also be considered as an additional potential deterioration pathway [178]. For bioactive delivery systems, active-compound retention and degradation kinetics should be regarded as critical quality attributes during shelf-life validation, as the loss of bioactive compounds can directly compromise the intended functionality of the product [179]. In addition, accelerated storage tests can be used to accelerate quality deterioration through stresses such as accelerated oxidation, while appropriate kinetic models can facilitate the prediction of stability under normal storage conditions and thereby shorten the time required for shelf-life validation [178].

## 7. Conclusions and Outlook

This review systematically reviews the stabilization mechanisms, particle design, gelation formation pathways, and application progress of food-grade Pickering emulsions and their gel systems. The irreversible adsorption of solid particles at the oil–water interfaces and steric hindrance effects, in synergy with the three-dimensional network of the continuous phase, endow the system with exceptional stability; particle wettability, size, morphology, and environmental factors collectively determine interfacial behavior and rheological properties. Protein, polysaccharide, and their composite particles provide rich regulatory means for interfacial stabilization and network construction, with composite particles demonstrating clear advantages in synergistically enhancing stability and integrating antioxidant and controlled-release functions. Based on the two gel formation modes—emulsion-filled gels and emulsion particulate gels—this system, by virtue of three-dimensional crosslinked networks and multiphase structures, has been successfully applied to simulating solid fat texture, protecting and delivering bioactive substances, constructing printable food inks, and active packaging materials. Nevertheless, the industrial translation of Pickering emulsion gels still faces challenges related to large-scale production, economic feasibility, regulatory approval, and shelf-life validation.

Future research on Pickering emulsions and their gel systems can be deepened in the following areas: First, the development of green, intelligent, and multifunctional Pickering emulsion gels. Using natural or modified natural solid nanoparticles as emulsifiers to prepare low-cost, environmentally friendly, and reusable Pickering emulsions; simultaneously, fully utilizing 4D printing technology to construct temperature, pH, magnetic field, and other intelligent responsive systems for slow release of substances, catalytic reactions, and other processes. Although such intelligent emulsions are currently mainly in the laboratory research stage, scholars worldwide have achieved numerous results in preparation methods and response mechanisms. Once technology matures and costs are controllable, broad application prospects will emerge.

Second, advancing research on targeted delivery and precision nutrition. The protection and delivery of functional components is transitioning from ‘roughly improving bioavailability’ to ‘precisely programmed sequential release.’ Achieving sequential release of different active substances at specific sites along the gastrointestinal tract at preset times in a delivery system is key to improving synergistic efficacy and bioavailability. In the latest research, Wang et al. have utilized solid–oil–water multilayer emulsions to construct a programmed sequential release system, enabling curcumin to target small intestinal release (67.3%) and fucoxanthin to target colonic release (60.3%), with bioavailability of the former improved by 5.4 times [180]. In the future, this can be further extended to combinations of active components requiring staged effects such as antioxidants and anti-inflammatory agents, prebiotics and postbiotics.

Finally, strengthening research on freeze–thaw applications. Frozen transport, cold chain storage, and thawing/reheating often cause irreversible changes in emulsion structure, such as compression damage caused by droplet shrinkage and oil crystallization growth [181]. How to improve the freeze–thaw cycle stability, ice crystal damage resistance, and post-thaw rheological recovery of Pickering emulsion gel systems is a key bottleneck for industrialized applications. Currently, stabilization strategies mainly include strengthening the interfacial anchoring of particles and forming dense viscoelastic interfacial films through enzymatic cross-linking, heat induction, glycosylation, or polyphenol complexation; optimizing the composition of the continuous phase (e.g., increasing the content of polysaccharides/KGM) to construct a three-dimensional gel network, while regulating interfacial and interparticle interactions through ionic strength and pH to restrict droplet migration and mitigate ice-crystal-induced compression; incorporating cryoprotective components (e.g., maltose and trehalose) to modulate the aqueous phase and alleviate ice-crystal-induced structural damage; and regulating particle concentration, oil-phase fraction, and freezing conditions to optimize interfacial coverage and ice-crystal formation behavior [43]. Future work needs to more deeply elucidate freeze–thaw destabilization mechanisms and accordingly develop personalized stabilization strategies and novel stabilization approaches for different scenarios, to promote large-scale industrialization of Pickering emulsion gels in the food industry.

## Figures and Tables

**Table 1:** Summary of protein-based particle types, sizes, shapes, principal applications and limitations.

Protein Class	Particle Type	Size	Shape	Primary Applications	Limitation	Reference
Protein Particles	Soy protein particles	100 nm	Spherical	Stabilizing O/W Pickering emulsions; developing high freeze–thaw stability emulsions.	A balance between freeze–thaw stability and flocculation needs to be achieved.	[51]
	Quinoa protein particles	132 nm	Near-spherical	Developing high-internal-phase emulsions (HIPEs) as potential replacements for partially hydrogenated oils.	High ultrasonic energy is required for effective nanoparticle assembly.	[52]
	Gelatin particles	200 nm	Spherical	Stabilizing HIPEs; encapsulating β-carotene, Encapsulation efficiency: 95%	—	[53]
	Lupin protein particles	100 nm	Approximately spherical	Stabilizing W/O Pickering emulsions.	—	[54,55]
	Peanut protein particles	178–260 nm	Approximately spherical	Stabilizing O/W Pickering emulsions; encapsulating and releasing 5-demethylnobiletin, bioaccessibility increased from 9.2% to 18.3%.	Particle assembly is sensitive to ionic strength.	[56]
	Whey protein particles	200–500 nm	Spherical	Stabilizing O/W Pickering emulsions.	The stability is significantly dependent on pH and ionic strength.	[57]
Protein Fibrils	Whey protein fibrils	Length: 200–1000 nm	Linear	Assembling hydrogels; encapsulating and protecting curcumin.	Poor stability caused by large droplets and low system viscosity.	[58]
	Ovotransferrin fibrils	Length: 150–550 nm (avg. 337 nm)	Filamentous	Stabilizing W/O Pickering emulsions.	—	[59]
Protein Microgels	Whey protein microgels	285–337 nm	Spherical	Stabilizing O/W Pickering emulsions; acting as stabilizers for triglyceride–water emulsions.	When the particles are insufficiently covered, bridging is likely to occur.	[60,61]
Protein Nanogels	Casein nanogels	179 nm	Spherical	Preparing O/W HIPEs.	The stability is significantly dependent on pH and ionic strength.	[62]

**Table 2:** Summary of polysaccharide-based particle types, sizes, shapes, principal applications and limitations.

Particle Type	Size	Shape	Primary Applications	Limitation	Reference
Native starch nanoparticles	251–453 nm	Spherical, ellipsoidal, polygonal	O/W Pickering emulsion stabilization	Sensitive to pH and salt concentration	[66]
OSA-modified starch particles	Avg. 33 nm	Spherical or rectangular	Encapsulation and controlled release of bioactive compounds (e.g., polyphenols); production of polymer and nanocomposite materials; food formulations for gluten-free rice bread and mayonnaise	An excessively high degree of OSA modification does not necessarily continue to improve stability.	[67,68]
Starch nanocrystals	43–396 nm	Ellipsoidal–spherical	Replacement of conventional emulsifiers; preparation of energy drinks, fermented beverages, and food supplements.	Acid hydrolysis required, and its in vivo safety remains to be evaluated	[69]
Starch nanospheres	270 nm	Spherical	Stabilizing Pickering emulsions as emulsifiers.	Sensitive to pH and water–oil ratio.	[70]
Cellulose nanocrystals (CNCs)	Diameter: 20 nm; length: >200 nm	Rod-shaped	Stabilizing O/W Pickering emulsions; use as antimicrobial materials.	The preparation process relies on acid hydrolysis, Stability is sensitive to the particle concentration and the proportion of oil.	[71]
Cellulose nanofibers (CNFs)	Length: >3000 nm; width: 33–64 nm	Filamentous	Stabilizing O/W Pickering emulsions as novel food-grade emulsifiers.	Preparation involves pre-treatment with NaOH/NaClO and high-pressure homogenization	[72,73]
Chitin nanocrystals	255 × 29 nm	Needle-shaped and partly spindle-shaped	Stabilizing O/W Pickering emulsions with internal phase volume fractions of 0.5–0.8; used in 3D-printed bio-inks exhibiting favorable textural properties.	Carboxylation requires oxidation with ammonium persulfate (APS)	[74]
Chitin nanofibers	343 × 13 nm	Fibrous, rod-shaped	Stabilizing O/W Pickering emulsions	The preparation process is usually energy-intensive, costly and has a significant environmental impact.	[75]
Chitosan nanoparticles	582.6 nm	Spherical	Stabilizing O/W Pickering emulsions; preparation of microcapsules by freeze-drying or spray-drying to extend shelf life	pH-dependent particle assembly	[76]
Hyaluronic acid nanoparticles	120 nm	Spherical	Stabilizing W/O Pickering emulsions; 3D-printing inks.	Multi-step synthesis may limit process simplicity and scalability.	[77,78]
Calcium alginate nanoparticles	316.5–412.9 nm	—	Preparing pH-responsive Pickering emulsions; Curcumin release: 37% (4 h, pH 6.8)	Hydrophobic modification and Ca^2+^ crosslinking are required for particle preparation.	[79]

**Table 3:** Summary of composite particle types, sizes, shapes, principal applications and limitations.

Composite Type	Particle Type	Size	Shape	Primary Applications	Limitation	Reference
Protein–Polysaccharide Composites	Ovotransferrin–gum arabic composite particles	175.8 ± 1.3 nm	Spherical	Stabilizing O/W Pickering emulsions; developing nutraceutical products.	Requires optimization of Ovotransferrin/gum arabic ratio and pH.	[81]
	Zein–chitosan composite particles	—	Spherical	Stabilizing O/W Pickering emulsions; encapsulating vitamin D3, Encapsulation efficiency: 91.8%	Sensitive to ionic strength and oil-phase content.	[82,83]
	Soy protein–chitosan composite particles	The average particle size is 148.52 ± 1.95 nm	—	Stabilizing O/W Pickering emulsions; Improving freeze–thaw stability of food systems.	Sensitive to particle concentration and water–oil ratio.	[84,85]
Polysaccharide–Polysaccharide Composites	Chitosan–starch composite nanogel particles	378.2 nm	Nanogel	Stabilizing O/W Pickering emulsions.	Requires optimization of chitosan–starch ratio and particle concentration.	[86]
	β-Cyclodextrin–glucan composite particles	104.48 nm	Spherical	Stabilizing O/W Pickering emulsions.	Requires optimization of particle composition and concentration.	[87]
	Cellulose–chitosan composite particles	22.38 ± 0.62 μm	Spherical	Stabilizing W/O Pickering emulsions; Fat replacement.	Emulsion type is sensitive to EC/CS ratio and water fraction.	[88,89]
Protein–Protein Composites	Ovotransferrin–lysozyme composite particles	—	Approximately spherical	HIPEs stabilizers; curcumin delivery with bioaccessibility increased from 16.1% to 38.3%	Sensitive to pH, protein ratio, and ionic strength.	[90]
	Soy protein–whey protein composite particles	122.4–153.3 nm	Polyhedral	HIPEs stabilizers.	Composition- and concentration-dependent performance.	[91]
	Zein–gum arabic composite particles	Avg. 225 nm	Spherical	Stabilizing high-oil-phase O/W Pickering emulsions.	Performance is sensitive to zein/GA ratio and oil fraction.	[92]
Protein–Polyphenol Composites	Zein–tannic acid composite particles	202.95 ± 7.05 nm	Spherical	Stabilizing O/W Pickering emulsions; Controlled release of curcumin	Requires optimization of zein/tannic ratio and tannic concentration.	[93,94]
Polysaccharide–Polyphenol Composites	Chitosan–caffeic acid composite particles	3500 nm	—	Stabilizing O/W Pickering emulsions; Developing bifunctional stabilizers with simultaneous stabilization and antioxidant properties.	Requires caffeic acid grafting; pH-dependent stability	[95]
Ternary Composites	Chitosan–sodium alginate–anthocyanin composite particles	220–665 nm	Spherical	Stabilizing O/W Pickering emulsions; Preparation and stabilization of antioxidant Pickering emulsions.	Particle concentration-dependent performance.	[96]
	Soy protein–citrus pectin–gallic acid composite particles	Avg. 1806.67 ± 133.87 nm	Spherical	Stabilizing HIPEs; Delivery carriers for β-carotene, Encapsulation efficiency: 95.97% ± 0.87%.	Performance depends on gallic acid concentration and oil fraction.	[97]
	PPI–gum arabic–gallic acid	307.27–315.67 nm	—	Stabilizing O/W Pickering emulsions; Encapsulation, protection and delivery of lycopene, Encapsulation efficiency: 92.8–96.7%	Particle aggregation occurs during thermal treatment, and particle assembly is sensitive to gallic acid concentration.	[98]

## Data Availability

No new data were created or analyzed in this study. Data sharing is not applicable to this article.
